# Metabolic profiling on the analysis of different parts of *Schisandra chinensis* based on UPLC-QTOF-MS with comparative bioactivity assays

**DOI:** 10.3389/fpls.2022.970535

**Published:** 2022-11-28

**Authors:** Jiushi Liu, Xinlu Mu, Jinmei Liang, Jianuo Zhang, Tingyan Qiang, Hongbo Li, Bin Li, Haitao Liu, Bengang Zhang

**Affiliations:** ^1^Key Laboratory of Bioactive Substances and Resources Utilization of Chinese Herbal Medicine, Ministry of Education, Institute of Medicinal Plant Development, Chinese Academy of Medical Sciences and Peking Union Medical College, Beijing, China; ^2^Department of Pharmacy, Medical Guarantee Center Pla General Hospital, Beijing, China; ^3^College of Horticulture, Shenyang Agricultural University, Shenyang, China

**Keywords:** *Schisandra chinensis*, different parts, metabolomics, bioactivity assays, chemometric

## Abstract

The *Schisandra chinensis* is an important edible plant, and previous phytochemical research focused on the *S. chinensis* fruit (SF) due to its long history as traditional Chinese medicine. *Schisandra chinensis* fruit was used as an astringent tonic to astringe the lungs and the kidneys, replenish energy, promote the production of body fluids, tonify the kidney, and induce sedation. The components of *S. chinensis*, such as its stems (SS), leaves (SL), and roots (SR), have drawn little attention regarding their metabolites and bioactivities. In this study, a strategy of combining a chemical database with the Progenesis QI informatics platform was applied to characterize the metabolites. A total of 332 compounds were tentatively identified, including lignans, triterpenoids, flavonoids, tannins, and other compound classes. Heatmap and principal component analysis (PCA) showed remarkable differences in different parts of the plants. By multiple orthogonal partial least-squares discriminant analyses (OPLS-DA), 76 compounds were identified as potential marker compounds that differentiate these different plant parts. Based on the variable influence on the projection score from OPLS-DA, the active substances including gomisin D, schisandrol B, schisantherin C, kadsuranin, and kadlongilactone F supported the fact that the biological activity of the roots was higher than that of the fruit. These substances can be used as marker compounds in the plant roots, which likely contribute to their antioxidant and anti-inflammatory activities. The plant roots could be a new medicinal source that exhibits better activity than that of traditional medicinal parts, which makes them worth exploring.

## Introduction

*Schisandra chinensis* is an important plant resource that is distributed in North China, D.P.R. Korea, R.O. Korea, Japan, and most Eastern parts of Russia; in addition, the plant has a very large market and offers significant economic and medicinal values (Panossian and Wikman, 2008; Ye et al., 2019). *Schisandra chinensis* is a special cash crop in rural areas of Northeast China, and the annual demand for *S. chinensis* as a traditional tonic herb and food is more than 30,000 tons. A key method for farmers to increase income and economic development was established by planting *S. chinensis* crops, and the planting area has expanded yearly. It is necessary to strengthen the research and utilization of *S. chinensis*, promote its industrial transformation, and upgrade the intensive method of processing. The *S. chinensis* fruit (SF), which is called Wuweizi in China, has been used for thousands of years in traditional Chinese medicine as a superior drug and has been included in the list of available healthy foods by the Ministry of Health of China since 2002 (http://www.nhfpc.gov.cn/sps/s3593/200810/bc239ea3d226449b86379f645dfd881d.html) (Medica Editorial Board of National Institute of Chinese Medicine, 1999). *Schisandra chinensis* fruit was used as an astringent tonic to astringe the lungs and the kidneys, replenish energy, promote the production of body fluids, tonify the kidney, and induce sedation (Chinese Pharmacopoeia Commission, 2020). Our ethnobotanical survey found that, aside from fruits, other parts of *S. chinensis* have been used in foods and herbal drugs for a long time, which contain nutrients and biologically active phytochemicals. Due to the lemon flavor that is usually absent in the most commonly used seasonings, local individuals in North China often dry *S. chinensis* stems (SS) and use them as a seasoning for stewed meat and as a substitute of pepper. The use of leaves and roots as traditional herbal tea is well known, and they have been used to delay the senescence process in Chinese folk medicine (Chen et al., 2011).

Gomisin A, schisandrol B, schisandrin, and schisandrin C are natural dibenzocyclooctadiene lignans that are isolated from SF and are considered to be the main active compounds responsible for the bioactivity of SF (Szopa et al., 2017). The compounds exhibit antihepatotoxic, antioxidant, antitumor activity, and anti-human immunodeficiency virus (HIV) activities as well as have effects on physical performance and the central nervous system (Xu et al., 2015; Chen et al., 2019; Yan et al., 2021). More recently, studies reported the therapeutic effects of *S. chinensis* on alleviating cough, liver injury, kidney injury, lung injury, platelet aggregation hepatitis, and cardiovascular disease (Gui et al., 2020; Xu et al., 2020; Lin et al., 2021). Gomisin M_1_ was found to modulate miRNA biogenesis to inhibit the proliferation, migration, and invasion of hepatocellular carcinoma (HCC) cells (Xu et al., 2021). Schisandrol B may play a role in the treatment of carbon tetrachloride (CCl_4_)-induced liver injury by downregulating the expression of iNOS and COX-2 and regulating the expression of the NF-κB and IL-17 signaling pathways to inhibit the expression of proinflammatory factors. Schisandrin B alleviated CCl_4_-induced liver inflammation and fibrosis by inhibiting macrophage polarization by targeting peroxisome proliferator-activated receptor gamma (PPARγ) (Chen et al., 2021). However, other parts of *S. chinensis*, such as its stems (SS), roots (SR), and leaves (SL), have been scarcely reported on their phytochemistry and bioactivity (Liu et al., 2020). Indeed, different parts of *S. chinensis* have different medicinal values; however, research on the chemical composition and biological activity of *S. chinensis* is limited, thus further development and utilization of this species are still challenging.

Because of the large number, structural diversity, and content range (10% to sub-ppm level) of chemical compounds in plants, performing secondary metabolomics analysis has been a great challenge (Hur et al., 2013; Lu et al., 2016). The development of liquid chromatography-tandem mass spectrometry (LC-MS) provides strong support for the metabolic profiling analysis of plant extracts (Chaleckis et al., 2019). Plant metabolomics was widely used in the analysis of metabolites from different geographical sources, different growth periods, and different plant parts (Dai et al., 2015; Han et al., 2015; Jandric et al., 2017). Until now, the use of ultra-performance liquid chromatography coupled with quadrupole/time-of-flight MS (UPLC-Q-TOF-MS/MS) to analyze *S. chinensis* samples has been used for only 28 marker compounds of SF extracts. Moreover, no report is available to compare the differential metabolites in the four parts of *S. chinensis*.

In the present study, the local database of metabolites from the genus *Schisandra* was established by using Progenesis SDF Studio. The metabolites of SL, SF, SS, and SR were compared, and the marker compounds were identified by UPLC-QTOF-MS coupled with chemometric analysis. Previous biological activity studies mainly focused on SF, and the results showed that SF exhibited antioxidant and anti-inflammatory activities in the cell line RAW 264.7 (Hu et al., 2012; Wang et al., 2018). The antioxidative and antiphlogistic are the basis for its liver protection activity and anti-neurodegenerative diseases (Guo et al., 2008). However, there have been no reports of antioxidant and anti-inflammatory activities with other parts of *S. chinensis*. Therefore, through screening the antioxidant and anti-inflammatory activities of different parts of *S. chinensis in vitro*, we may find alternative sources of the antioxidants and anti-inflammatory compounds; thus, we can study the correlation between chemical composition and biological activities in the plant parts of *S. chinensis*.

## Materials and methods

### Materials

The four different parts, SF, SR, SS, and SL, were collected from plants in Shenyang, Liaoning province, China, in September 2020. The certificate specimen was identified by Professor Bengang Zhang from the Institute of Medicinal Plant Development (IMPLAD) and deposited at the Medical Plant Resource Center in the IMPLAD.

### Chemicals and reagents

Reference substances were used to compare the MS data, and retention time (RT) of the identified compounds consist of schisandrol A, pregomisin, schisantherin B, schisantherin A, schisantherin D, schisandrin B, benzoylgomisin O, interiotherin A, angeloylgomisin O, gomisin D, angeloylisogomisin O, schisanhenol, schisandrol B, gomisin J, gomisin O, gomisin G, gomisin K_2_, gomisin K_3_, schisandrin C, schisandrin A, and gomisin N. All of the above compounds were isolated from *S. chinensis* and stored in the laboratory. For UPLC-MS analysis, LC/MS-grade acetonitrile, methanol, and formic acid were purchased from Thermo Fisher (USA), and water was purchased from Guangzhou Watsons Food & Beverage Co., Ltd. (GuangZhou, China). The dimethyl sulfoxide (DMSO) and 2,2′-diphenyl-1-picrylhydrazyl (DPPH) were purchased from Solarbio, Beijing, China. Then, 96-well plates were purchased from Promega Corporation (Madison, WI, USA). The analytical grade of methanol was procured from Beijing Chemical Works Beijing, China). Pure water (18.2 MΩ) was obtained from a Milli-Q System (Millipore, Billerica, MA, USA). Dulbecco's modified Eagle medium (DMEM), fetal bovine serum (FBS), and Cell Counting Kit-8 (CCK-8) were purchased from Gibco Life Tech (Waltham, MA, USA) and Beyotime Bio-Technology Co., Ltd. (Shanghai, China). Lipopolysaccharide (LPS) was purchased from Sigma Aldrich Corporation (St. Louis, USA). Nitric Oxide Assay Kit was obtained from the Beyotime Institute of Biotechnology (Shanghai, China).

### Sample preparation

The fruit, root, stem, and leaf samples were air dried, powdered, and sifted through a 50-mesh sieve. The extraction method in Chinese Pharmacopoeia 2020 was applied: each powdered sample (0.25 g) was added into a 20 ml volumetric flask, methanol was poured, sonication treatment was carried out for 30 min (250 W, 20 KHz), and the sample solution was filtered through a 0.22 μm membrane filter prior to injection into the UPLC-QTOF-MS system. Each part has three samples, and every sample was repeated two times. The stability and repeatability of the methodology, which employed gradient elution, were determined by the repeat analysis of a pooled quality control (QC) sample which was mixed with the 24 samples of *S. chinensis*. Finally, the filtrate was diluted 10-fold with methanol for the UPLC-QTOF-MS analysis.

### UPLC-QTOF/MS conditions

Ultra-performance liquid chromatography was performed on a Waters Acquity UPLC system (Waters, Milford, MA, USA), equipped with a diode array detection (DAD) system, which was recorded in the range of 200–400 nm. A Waters ACQUITY BEH C18 Column (2.1 × 100 mm, 1.7 μm, Waters, Milford, MA, USA) was applied for UPLC separation. Phase A was water and phase B was acetonitrile. The UPLC gradient elution was applied as follows: 49–49% B (0–2 min), 49–52% B (2–4 min), 52–60% B (4–6 min), 60–62% B (6–9 min), 62–62% B (9–10 min), 62–66% B (10–13 min), 66–70% B (13–15 min), and 70–95% B (15–17 min). The flow rate was 0.3 mL/min, and the injected volume of the sample solution was 1 μl. The column and autosampler were maintained at 30 and 20°C, respectively.

A Waters Xevo G2 QTOF (Waters, Milford, MA, USA) was used for MS detection. The instrument equipped with an electrospray ionization (ESI) source controlled by MassLynx 4.1 software. The conditions of the MS detector were as follows: capillary of 3 kV, sampling cone of 30 V, source temperature of 100°C, desolvation temperature of 300°C, cone gas flow of 50 L/h, desolvation gas flow of 600 L/h, and collision energy of 6 kV. Then, nitrogen was used as a nebulizer and auxiliary gas. MS^E^ data were obtained in centroid mode with a mass range of 100–1,200 Da, and the scanning time is 1 s. The instrument was performed in both low-energy (function 1) and high-energy (function 2) scan functions, and the collision energy was 6 and 20–40 V, respectively. The lock mass compound was 200 pg/μL of leucine enkephalin (*m/z* 556.2771 in positive mode).

### Main lignans content detection

We detected the following 9 common lignans in *S. chinensis* and investigated their distribution in different parts: schisandrol A (1), gomisin D (2), gomisin J (3), schisandrol B (4), angeloylgomisin H (5), gomisin G (6), schisantherin A (7), schisandrin B (8), and schisandrin C (9). The extract method and methodology for content detection were determined according to Mu et al. (2022). A sample (1 g) was mixed with 30 mL of methanol in a capped conical flask and was extracted three times by using the ultrasonic method (60 kHZ, 250 W), for 20 min each time. The weight loss in the ultrasonic procedure was compensated and filtered through a 0.22-μm membrane filter prior to the UPLC system. The chromatographic method is the same as the above “2.4 UPLC-QTOF/MS conditions.”

### Construction of the *Schisandra* compound database

A local database of the genus *Schisandra* was created using Progenesis SDF Studio. The compounds from *Schisandra* species were sorted and summarized by searching SciFinder, Pubmed, CNKI, and Google Scholar. The CAS number was obtained by searching SciFinder for its name or structure (found in the original article) and verifying the single structure file (·mol) of the selected compound and transfer. All ·mol files were consolidated into an internal database by Progenesis SDF Studio. The established database was used to identify the compounds.

### Data processing and analysis

The data were profiled using Progenesis QI 2.3 software, which was obtained from positive ion modes of different parts (Waters, USA). The operation steps by Progenesis QI are as follows: data import, peak alignment, experimental grouping, peak extraction, normalization, deconvolution, identification, and statistical analysis. The 30 runs imported were calibrated according to automatically selected QC samples. Peak extraction setting parameters were as follows: limits automatic, sensitivity default set to 3, chromatographic minimum peak width, and 1.0–17.0 min of retention time (RT). Structures of the chemical constituents were identified by the *Schisandra* compound database, which was constructed by a ·mol file in Progenesis SDF Studio. In addition, an online compound library (Nature Chemical Biology) was used to identify more chemical compounds. The adduct ion in Progression QI 2.3 was setted as follows: [M+H]^+^, [M+Na]^+^, [M+K]^+^, [M+NH_4_]^+^, [2M+H]^+^, and [2M+Na]^+^. The data were imported in a .raw format, and chromatographic peak alignment was used to set RT limits, including ignoring ions before 1 min and after 17 min; then, the automatic processing was completed, the experimental design was setup, the compound identification was carried out, and compound statistics were applied. SIMCA 14.1 was employed to process exported data, using principal component analysis (PCA) and orthogonal partial least-squares discriminant analysis (OPLS-DA), combined with S-plot to analyze potential marker compounds. In S-plots, the significant differential retention time-exact mass (RT-EM) pairs were chosen and marked on the Progenesis QI for compound identification. The RT-EM pairs were finally determined by variance analysis, with a *p*-value of ≤ 0.05 and a max fold change of ≥2.

### Antioxidant activities

To evaluate the DPPH radical scavenging activity, the Solarbio Kit was used. Following the method (Usman et al., 2022), 10.0000 mg from different parts were accurately weighed, 10 ml of extract solution was added and extracted in a 40°C water bath for 30 min, and then, the gradient was diluted to 1.000, 0.5000, 0.2500, 0.1250, 0.0625, and 0.0313 mg/ml. After centrifugation at room temperature of 10,000 rpm for 10 min, the supernatant was put on ice for testing. The following reagents were added into a 1.5 ml EP tube: blank tube (25 μl extract + 975 μl working solution), determination tube (25 μl supernatant + 975 μl working solution), and control tube (25 μl supernatant + 975 μl absolute ethanol); DPPH free radical scavenging rate is defined as D% = {[A blank − (A determination − A control)]/A blank} ^*^ 100%.

### Anti-inflammatory activities

Cell culture and the determination of cell viability was the first step to assay the anti-inflammatory activities of different parts. RAW 264.7 macrophages were purchased from the cell bank of the Type Culture Preservation Committee of the Chinese Academy of Science (Shanghai, China) and were selected to verify cytotoxicity. Then, to measure the content of Nitric oxide (NO), the NO standard curve was drawn and determined by the Griess kit. The specific methods are shown in Supplementary material 1.

Finally, the appropriate dose and concentration were selected according to cell viability to determine NO. To establish the inflammatory cell model, a total of 200 μL of RWA264.7 cells (2 × 106 cells/mL) were added into 96-well plates and cultured at 37°C in 5% CO_2_ for 24 h. Next, 100 μL of different concentrations extracts (25, 50, 100, and 200 μg/mL) from SR, SS, SL, and SF were added to the culture plate and incubated for 1 h. Afterward, 1 μg/mL of LPS was added to the culture plate and incubated for 24 h, and the supernatant was obtained. Finally, 50 μL of supernatant, 50 μL of Griess Reagent I, and 50 μL of Griess Reagent II were added to the culture plate, and the absorbance at 540 nm was determined by a microplate reader. The NO concentration of each group was calculated according to the obtained standard curve.

## Results and discussion

### The database of the genus *Schisandra*

A total of 237 compounds, including lignans, triterpenoids, flavonoids, tannins, and other compound classes, were searched from the phytochemistry and pharmacology literature of the genus *S. chinensis* (last updated December 2021). The database has been the most complete MS database for the genus *Schisandra* thus far. The *Schisandra* compound database included an additional 308 compounds from *Schisandra sphenanthera* and other species, in which many studies on phytochemistry as traditional Materia medica were performed (Szopa et al., 2019; Yang and Yuan, 2021; Yang et al., 2021). Thus, the 545 compounds were confirmed, and the ·mol files were stored.

The *Schisandra* compound database included 545 compounds from *Schisandra*, and each compound contained the following information: scientific name, CAS number, neutral mass, formula, and structure. The *Schisandra* compound database is conducive to the metabolic identification of *S. chinensis* and promotes the characterization of compounds from other *Schisandra* species. A new research model can be used as a reference to establish personalized databases for other species, and the size of these chemical databases may vary greatly because of the number of species.

### Metabolite characterization and compound identification

Based on the local database, which was premade, and the online compound library, 332 compounds were detected by combining the Progenesis MetScope in Progenesis QI, including lignans, flavonoids, triterpenoids, and alkaloids. A total list of detected compounds, which consists of the formula and adducts, is available in the Supplementary material (Table S1). Lignans are the most important compounds in the genus *Schisandra*, especially dibenzocyclooctene lignans, such as schisantherrin A, schizandrin, deoxyschizandrin, γ-schizandrin, and gomisin J (Szopa et al., 2017). Most dibenzocyclooctene lignans show a variety of biological activities, including anti-inflammatory, antioxidant, antihepatotoxic, and antitumor activities. Several other compounds, including triterpenoids, flavonoids, tannins, and precursor organic acids, were also identified.

**Figure 1:**
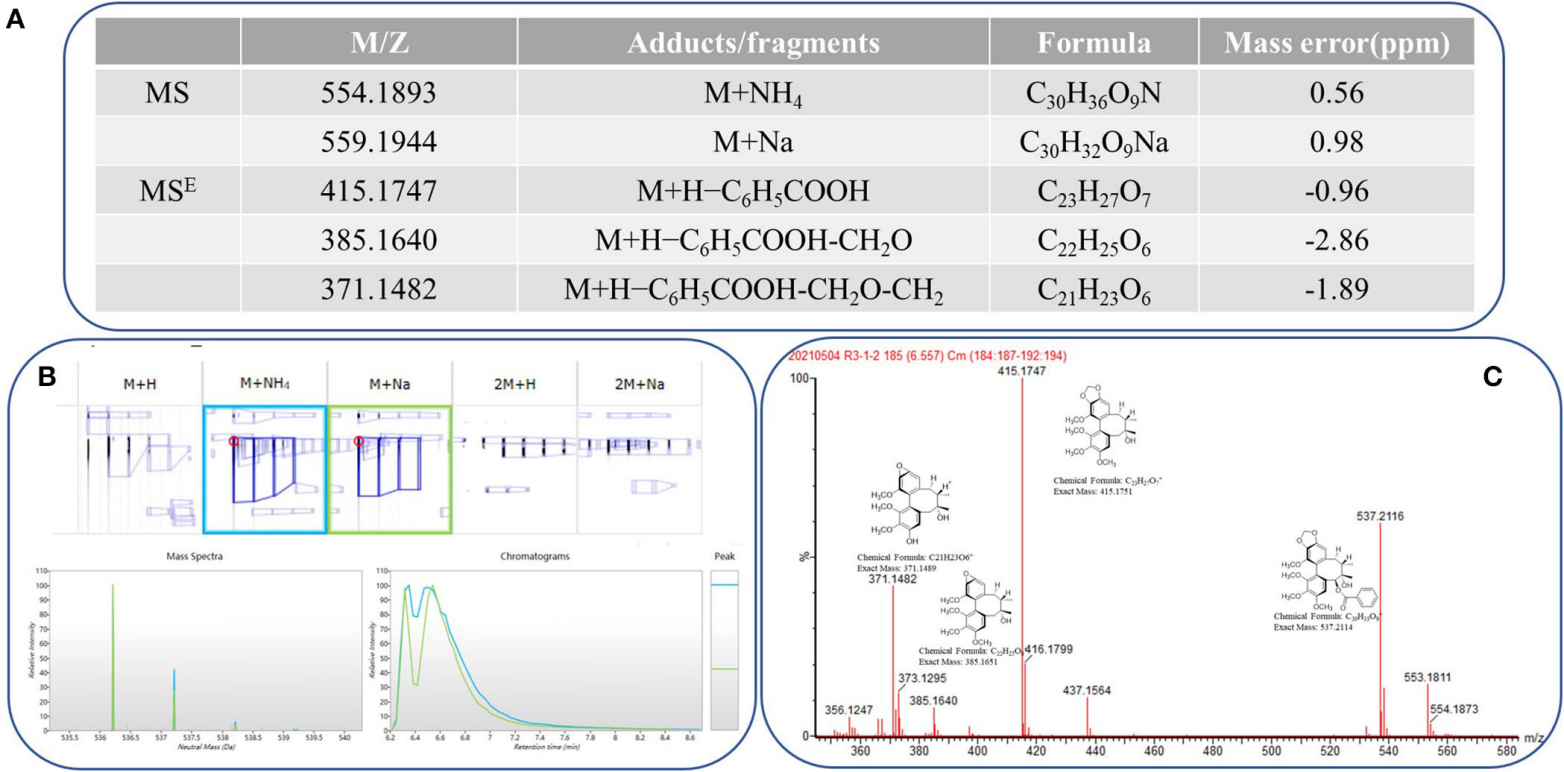
Progenesis QI showing the identification of schisantherin A from the filtered metabolic features. **(A)** Low-energy major precursor exact mass schisantherin A adduct ions and corresponding high-energy fragment ions. **(B)** Common adduct ions for schisantherin A. **(C)** Structure and exact mass number of fragment ions.

A total of 30 UPLC-MS samples were imported to Progenesis QI for data analysis. The ion at 416.1839 *m/z* (RT 3.51 min) was identified as a reliable example. A strong peak of [M+Na]^+^ (*m/z* 559.1979) was observed during the full scan MS at 6.56 min, which is characterized by a loss of neutral molecules, such as C_6_H_5_COOH (−122 Da), OCH_2_ (−30 Da), and H_2_O (−18 Da), and the presence of some fragment ions, including [M+H-H_2_O]^+^, [M+H-C_6_H_5_COOH]^+^, and [M+H-C_6_H_5_COOH-CH_2_O]^+^. Finally, the compound was identified as schisantherin A, and the fragmentation is shown in Figure 1C. These fragments corresponded with inferred Progenesis QI and the standard ion fragment (Figure 1).

### Chemical comparison and biomarker probe between different parts

**Figure 2:**
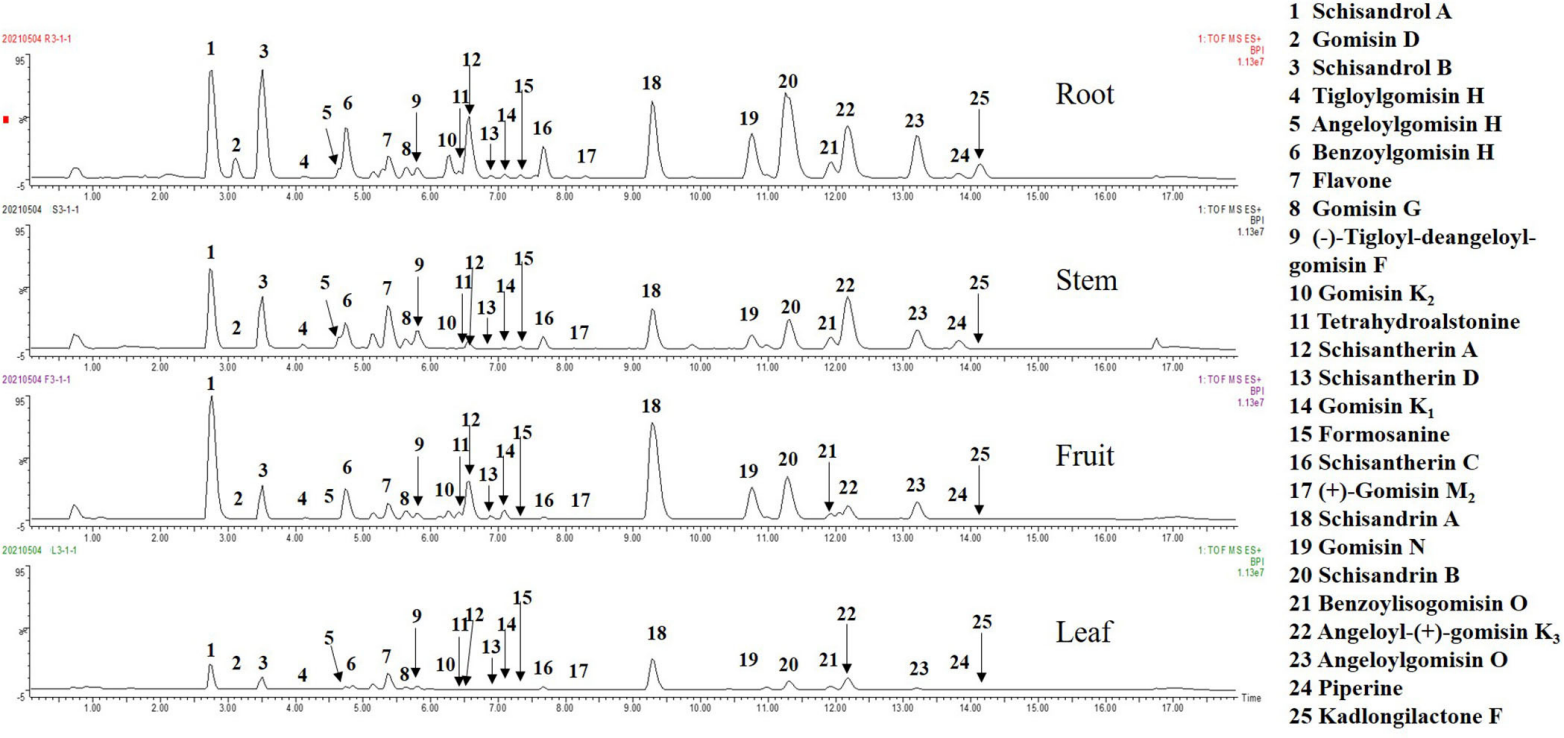
The basic peak ion (BPI) chromatograms in positive ion mode.

To study the metabolic diversity of different parts of *S. chinensis*, the extracts of fruits, leaves, stems, and roots were comparatively studied by UPLC-QTOF-MS. The basic peak ion (BPI) chromatogram of fruits, leaves, stems, and roots revealed significant differences in their overall composition (Figure 2). To fully understand the chemical differences between samples, all compounds were used for chemometrics without any filtration.

**Figure 3:**
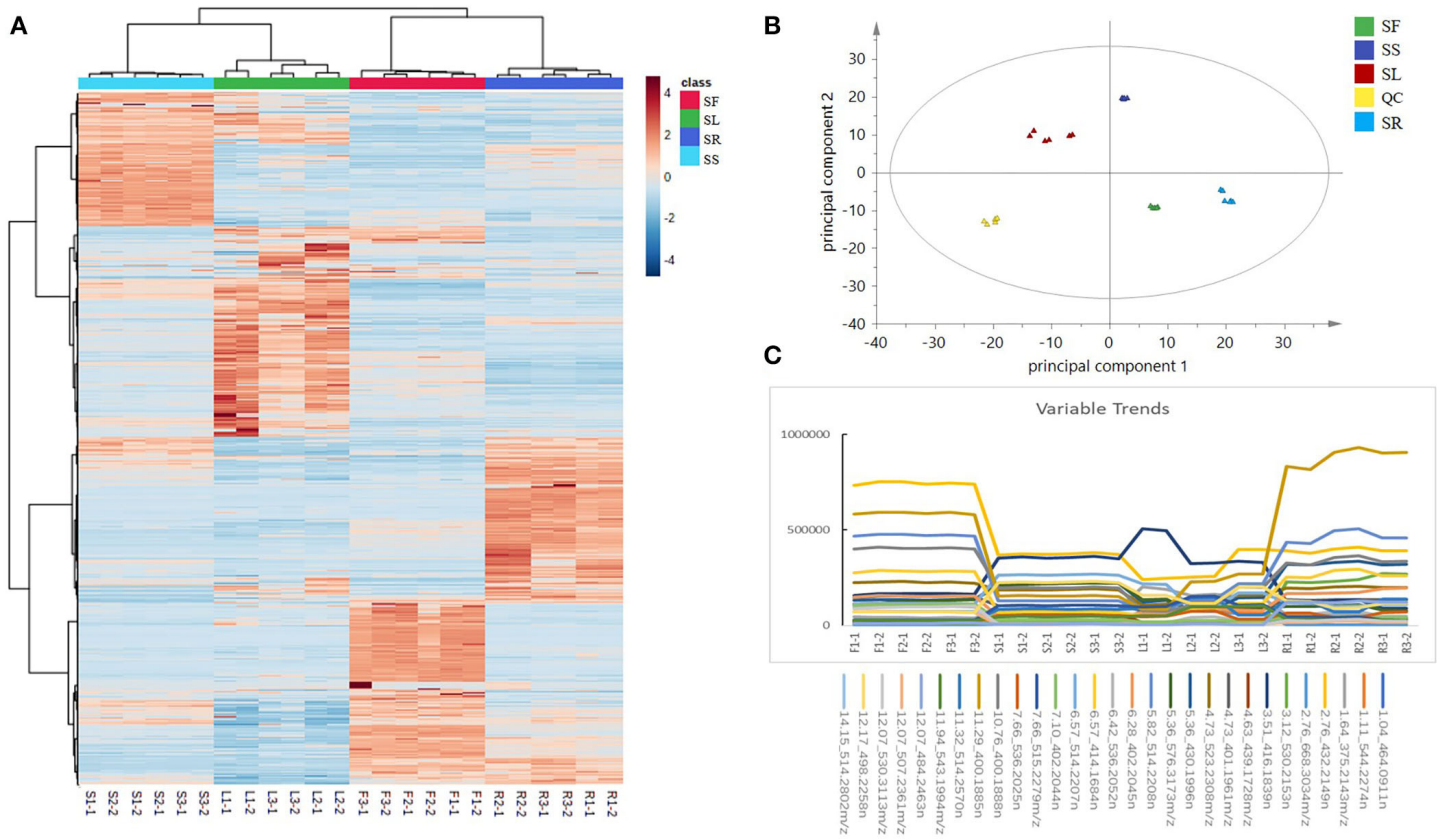
**(A)** A heatmap based on the relative abundance of 3,302 identified compounds in different parts of *Schisandra chinensis*. **(B)** A PCA score plot of five parts of *S. chinensis*: stem (SS), leaf (SL), fruit (SF), root (SR), and quality control (QC). **(C)** Examples of variation trends of different compounds in different parts of *S. chinensis*.

To understand the distribution characteristics of all compounds in different sections, heatmapping, the most common visualization method is widely used in metabonomics because of its vivid information expression (Trygg et al., 2007). It is worth noting that all peaks detected by QI in the heatmap have remarkable differences in different parts, and few metabolites had a higher relative content in all parts of the same plant. In addition, the clustering results showed that there was significant diversity among groups but almost no differences within groups (Figure 3A). The peaks varied greatly among different parts; for example, the content of deoxyschizandrin was in the order of SF > SR > SS > SL and of γ-schizandrin was SR > SF > SS > SL.

The PCA is a multivariate statistical analysis method that uses the linear transformation of multiple variables to select fewer significant variables. It is widely used in metabonomics to find the molecules that cause the differences between samples or groups and to further analyze the metabolic pathways, biomarkers, and biological significance (Lever et al., 2017). The QC samples were gathered by a PCA score plot (Figure 3B). There were significant discrepancies between the different parts, and except for within the SL group, the degree of polymerization was good among the groups, which was consistent with the heatmap results. The PCA score plot showed that the distance between roots and leaves was the farthest, which indicated that the divergence between them was the most obvious, which was consistent with the result of the BPI chromatogram. Obvious differences were observed in the metabolic spectrum of each part of *S. chinensis* by the PCA. The selected markers showed different variable trends between the different parts of *S. chinensi*s (Figure 3C). Leaves, roots, stems, and fruits have different chemical constituents. For instance, the contents of gomisin D (530.2153n, RT 3.12 min), schisandrol B (416.1839n, RT 3.51 min), and schisantherin C (515.2279n, RT 7.66 min) were higher in roots, while the contents were lower in leaves and stems. The difference in the metabolite spectrum may also help to explain the different efficacies of different parts of *S. chinensis*.

**Figure 4:**
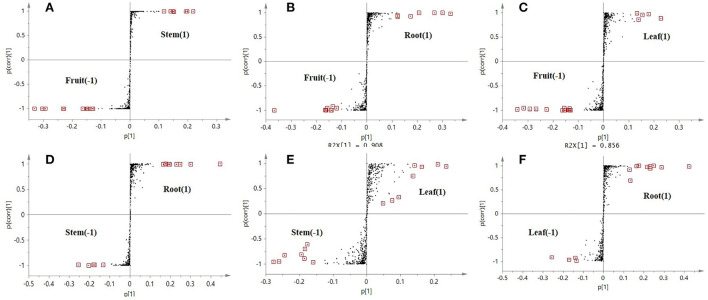
S-plot score from the six different comparison models along with selected candidate marker compounds from each model **(A–F)**.

Orthogonal partial least-squares discriminant analysis was used to perform further discriminant analysis of metabolomics data in the two groups (Brereton, 2009). Four groups were compared by OPLS-DA with an S-plot to find marker compounds representing the difference between groups. These four groups were compared by OPLS-DA with an S-plot to further identify marker compounds causing the difference among groups. The comparison of parameters and models is shown in Table 1. Each model was reliable according to high R2 and Q2 values. The S-plot has a higher research value and shows the observation variables clearly on the two-dimensional plane. It was conducive to screening the correlation between chemical composition and the model type. The variable correlation and contributions were shown separately on the X- and Y-axes. The variable results displayed distinct differences among the groups and were found at the top right (1) and the bottom left (−1), and the ions with no significant difference were in the middle of the S-plot. Six model classes were applied to screen the biomarkers (Figure 4). The potential biomarkers were screened by the ions at both ends of the S-plot (the red box marked in Figure 4).

**Table 1:** Statistical parameter values of different orthogonal partial least-squares discriminant analysis (OPLS-DA) models based on ultra-performance liquid chromatography coupled with quadrupole/time-of-flight mass spectrometry (UPLC-Q-TOF-MS/MS) data in positive mode and the number of markers selected from S-plot.

	**OPLS-DA**				**S-plot**	
**Model classes**	**Scaling**	**Components**	**R2 (cum^a^)^b^**	**Q2 (cum)^c^**	**Markers**	**Markers**
			**(%)**	**(%)**	**in –1**	**in 1**
F vs. S	Pareto	2	0.991	1.000	9	8
F vs. R	Pareto	2	0.908	0.990	14	6
F vs. L	Pareto	2	0.929	0.994	14	5
S vs. R	Pareto	2	0.954	0.994	5	8
S vs. L	Pareto	2	0.992	0.988	8	8
R vs. L	Pareto	2	0.905	0.995	4	10

a cum: cumulative.

b R2 (cum): the variation displayed by all components in the model.

c Q2 (cum): the accuracy of the predicted class membership by the model.

**Figure 5:**
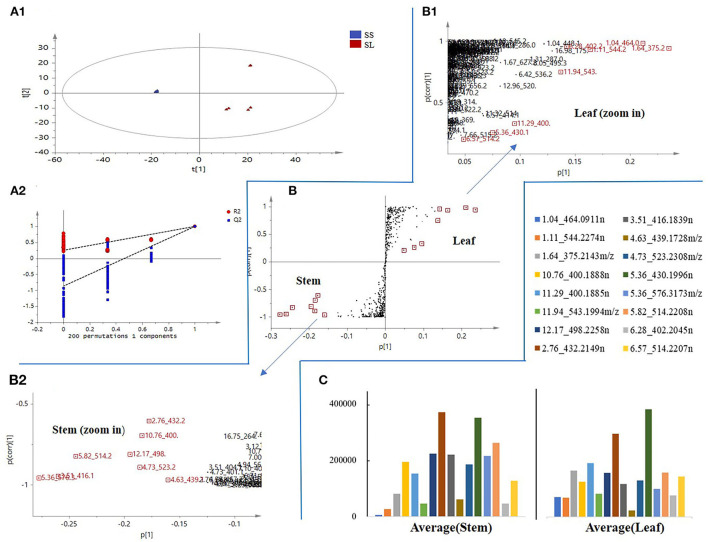
An example of marker compound selection in *S. chinensis*. **(A)** orthogonal partial least-squares discriminant analysis (OPLS-DA) score plot and the reliability of the OPLS-DA model. **(B)** S-plot score of SS and SL. **(C)** The trend of the relative contents of different compounds in SS and SL.

The results showed that 193 candidate marker compounds were screened and marked. These marker compounds were introduced into Progenesis QI for further identification. Of 193 compounds, 76 marker compounds were inferred by filtering defining parameters with an ANOVA *p*-value of ≤ 0.05 and a max fold change of ≥2 and retrieving from multiple databases (Table 2). Furthermore, 54 of 76 compounds were reported from other *Schisandra* species, and 9 compounds were identified for the first time from *Schisandra*. An example in Figure 5 shows that the marker compounds were identified. The S-plot score of the comparison between SS and SL is shown in Figure 5B. The screened marker ions of the top S-plot are enlarged in Figure 5B1, and the screened ions of the bottom S-plot are displayed in Figure 5B2. The trends of the content variable are shown in Figure 5C. The averages of the selected markers showed the differences between SS and SL.

**Table 2:** A total of 76 metabolites identified in positive ion mode.

**No**.	**RT-EM**	**Tentative identification**	**Adducts**	**Formula**	**Mass error (ppm)**	**UPLC-QTOF-MS**			
						**F**	**L**	**S**	**R**
1	1.04_371.1627*m/z*	(+)-Chelidonine	M+NH_4_	C_20_H_19_NO_5_	5.10	√	√	√	√
2	1.04_448.1017n	Plantaginin	M+Na, 2M+Na, M+H	C_21_H_20_O_11_	1.04	√	√	√	√
3	1.04_464.0911n	Quercetin-3-O-glucoside	M+Na, 2M+Na, M+H	C_21_H_20_O_12_	−6.42		√		
4	1.04_579.1526*m/z*	Procyanidin B_2_	M+H	C_30_H_26_O_12_	5.03	√	√	√	√
5	1.11_544.2274n	Schindilactone C	M+Na, 2M+Na, M+NH_4_	C_29_H_36_O_10_	−6.25		√		
6	1.60_819.4292*m/z*	Mitragynine	2M+Na	C_23_H_30_N_2_O_4_	−1.43	√	√	√	√
7	1.64_375.2143*m/z*	Schineolignins B	M+H	C_22_H_30_O_5_	−6.22		√		
8	1.70_328.1397*m/z*	N-Fructosyl phenylalanine	M+H	C_15_H_21_NO_7_	1.79	√	√	√	√
9	1.70_364.1524n	Coralyne	M+H, 2M+H	C_22_H_22_NO_4_	−6.92	√	√	√	√
10	1.84_657.2447*m/z*	Piperlongumine	2M+Na	C_17_H_19_NO_5_	4.48	√	√	√	√
11	2.43_733.3994*m/z*	Hirsuteine	2M+H	C_22_H_26_N_2_O_3_	4.65	√	√	√	√
12	2.76_284.0696n	Wogonin	2M+H, 2M+Na, M+H, M+Na	C_16_H_12_O_5_	3.99	√	√	√	√
13	2.76_432.2149n	Schisandrol A^*^	M+H, M+NH_4_, 2M+Na	C_24_H_32_O_7_	0.14			√	√
14	2.76_668.3034*m/z*	Aconitine	M+Na	C_34_H_47_NO_11_	−1.10	√			
15	3.12_530.2153n	Gomisin D^*^	M+Na, 2M+Na, 2M+H, M+NH_4_, M+H	C_28_H_34_O_10_	0.12	√	√	√	√
16	3.22_497.2856*m/z*	Atractylenolide III	2M+H	C_15_H_20_O_3_	−6.39	√	√	√	√
17	3.29_389.1980*m/z*	Gomisin J^*^	M+H	C_22_H_28_O_6_	−5.97	√	√	√	√
18	3.29_580.1782n	Naringin	M+NH_4_, M+Na	C_27_H_32_O_14_	−1.66	√	√	√	√
19	3.51_254.0595n	Daidzein	M+H, 2M+H, M+NH_4_	C_15_H_10_O_4_	6.36	√	√	√	√
20	3.51_416.1839n	Schisandrol B^*^	M+Na, 2M+Na, M+NH_4_	C_23_H_28_O_7_	0.98			√	√
21	4.14_523.2269*m/z*	Tigloylgomisin H	M+Na	C_28_H_36_O_8_	−6.67	√	√	√	√
22	4.31_390.2037n	Pregomisin^*^	M+H, M+Na	C_22_H_30_O_6_	−1.44	√	√	√	√
23	4.63_240.0872*m/z*	Isofraxidin	M+NH_4_	C_11_H_10_O_5_	2.69	√	√	√	√
24	4.63_357.1353*m/z*	3-Methoxycinnamic acid	2M+H	C_10_H_10_O_3_	5.79	√	√	√	√
25	4.63_439.1728*m/z*	5,6,7,8-Tetrahydro-1,2,3,13-tetramethoxy-6,7-Dimethylbenzo[3,4]cycloocta[1,2-f][1,3]benzodioxol-5-ol	M+Na	C_23_H_28_O_7_	0.23			√	√
26	4.73_401.1961*m/z*	Kadsuranin	M+H	C_23_H_28_O_6_	0.54	√			√
27	4.73_523.2308*m/z*	Angeloylgomisin H^*^	M+Na	C_28_H_36_O_8_	1.08	√		√	√
28	4.94_522.2256n	Benzoylgomisin H	M+Na, 2M+Na, M+NH_4_	C_30_H_34_O_8_	0.48	√	√	√	√
29	5.36_206.0599n	Scoparone	M+Na, 2M+Na	C_11_H_10_O_4_	5.78	√	√	√	√
30	5.36_223.0743*m/z*	Flavone	M+H	C_15_H_10_O_2_	−4.86	√	√	√	√
31	5.36_225.0905*m/z*	Flavanone	M+H	C_15_H_10_O_2_	−2.25	√	√	√	√
32	5.36_430.1996n	Schisanchinins C	M+H, M+Na, 2M+H, 2M+Na	C_24_H_30_O_7_	0.96		√	√	
33	5.36_576.3173*m/z*	Propindilactone J	M+NH_4_	C_31_H_42_O_9_	1.09			√	
34	5.40_727.3490*m/z*	Gelsevirine	2M+Na	C_21_H_24_N_2_O_3_	3.43	√	√	√	√
35	5.62_536.2053n	Gomisin G^*^	M+Na, M+NH_4_	C_30_H_32_O_9_	1.33	√	√	√	√
36	5.82_514.2208n	(-)-Tigloyl-deangeloyl-gomisin F	M+Na, 2M+Na, M+NH_4_	C_28_H_34_O_9_	1.03			√	
37	6.28_402.2045n	Gomisin K${}_{2}^{*}$	M+H, M+NH_4_, M+Na, 2M+Na	C_23_H_30_O_6_	0.66	√			√
38	6.38_993.3792*m/z*	Tetrahydroalstonine	2M+H	C_26_H_28_N_2_O_8_	2.78	√	√	√	√
39	6.56_536.2052n	Schisantherin A^*^	M+Na, 2M+Na, M+NH_4_	C_30_H_32_O_9_	0.98	√			√
40	6.56_654.2913*m/z*	Mesaconitine	M+Na	C_33_H_45_NO_11_	4.44	√	√	√	√
41	6.57_271.0599*m/z*	Baicalein	M+H	C_15_H_10_O_5_	−0.75	√	√	√	√
42	6.57_414.1684n	Kadsulignan L	M+H, M+Na, 2M+H	C_23_H_26_O_7_	1.21	√			√
43	6.57_514.2207n	Tigloylgomisin P	M+Na, 2M+Na, M+NH_4_	C_28_H_34_O_9_	0.83	√			√
44	6.57_616.3108*m/z*	Hypaconitine	M+H	C_33_H_45_NO_10_	−1.28	√	√	√	√
45	6.71_543.1633*m/z*	Schisantherin D^*^	M+Na	C_29_H_28_O_9_	1.38	√	√	√	√
46	6.71_559.1378*m/z*	Formononetin	2M+Na	C_16_H_12_O_4_	2.66	√	√	√	√
47	6.78_364.1502*m/z*	Isocorydine	M+Na	C_20_H_23_NO_4_	−5.06	√	√	√	√
48	7.10_402.2044n	Gomisin K_1_	M+H, M+Na, M+NH_4_, 2M+Na	C_23_H_30_O_6_	0.23	√			
49	7.20_425.1457*m/z*	Ginkgolide B	M+H	C_20_H_24_O_10_	3.52	√	√	√	√
50	7.34_759.3292*m/z*	Formosanine	2M+Na	C_21_H_24_N_2_O_4_	−5.76	√	√	√	√
51	7.56_359.1485*m/z*	Matairesinol	M+H	C_20_H_22_O_6_	−1.06	√	√	√	√
52	7.66_515.2279*m/z*	Schisantherin C	M+H	C_28_H_34_O_9_	0.83		√		√
53	7.66_536.2025n	Gomisin C	M+H, M+Na	C_30_H_32_O_9_	−4.01		√		
54	7.99_386.1728n	(+)-Gomisin M_1_	M+H, M+Na, 2M+Na	C_22_H_26_O_6_	−2.10	√	√	√	√
55	7.99_653.4038*m/z*	Ajmaline	2M+H	C_20_H_26_N_2_O_2_	−3.57	√	√	√	√
56	8.29_386.1721n	(+)-Gomisin M_2_	M+H, M+Na, 2M+Na	C_22_H_26_O_6_	−2.10	√	√	√	√
57	9.06_833.4501*m/z*	Schisandrin A^*^	2M+H	C_24_H_32_O_6_	3.65	√	√	√	√
58	10.76_284.0696n	Acacetin	M+H, 2M+Na	C_16_H_12_O_5_	3.85	√	√	√	√
59	10.76_290.0927n	Biochanin A	M+H, 2M+Na	C_16_H_12_O_5_	3.85	√	√	√	√
60	10.76_400.1888n	Gomisin N^*^	M+H, M+Na, 2M+Na	C_23_H_28_O_6_	0.52	√			√
61	11.29_228.0795n	3,4,5-Trihydroxystilbene	M+H, M+Na, 2M+H	C_14_H_12_O_3_	3.69	√	√	√	√
62	11.29_400.1885n	Schisandrin B^*^	M+H, M+Na, M+NH_4_, 2M+Na	C_23_H_28_O_6_	0.87	√			√
63	11.32_514.2570n	Kadsufolin A	M+Na, 2M+Na, M+NH_4_	C_29_H_38_O_8_	0.71		√	√	
64	11.94_543.1994*m/z*	Benzoylisogomisin O	M+Na	C_30_H_32_O_8_	0.72		√		
65	12.07_484.2463n	Angeloyl-(+)-gomisin K${}_{3}^{*}$	M+H, M+NH_4_, 2M+Na	C_28_H_36_O_7_	0.28	√			
66	12.07_507.2361*m/z*	Xuetongdilactone E	M+Na	C_28_H_36_O_7_	1.66	√			
67	12.07_530.3113*m/z*	Schinchinenins E	M+NH_4_	C_30_H_40_O_7_	0.17	√			
68	12.17_498.2258n	Angeloylisogomisin O^*^	M+Na, 2M+Na	C_28_H_34_O_8_	0.82		√	√	
69	12.60_329.1769*m/z*	Anwuligan	M+H	C_20_H_24_O4	6.71	√	√	√	√
70	12.60_372.1572n	Arctigenin	M+H, M+Na	C_21_H_24_O_6_	−0.19	√	√	√	√
71	12.60_384.1557n	Schisandrin C^*^	M+H, M+Na, 2M+Na	C_22_H_24_O_6_	−4.19	√	√	√	√
72	12.96_520.2100n	Benzoylgomisin O^*^	M+Na, 2M+Na, M+NH_4_	C_30_H_32_O_8_	0.59	√	√	√	√
73	13.06_505.1838*m/z*	Interiotherin A^*^	M+H	C_29_H_28_O_8_	−3.67	√	√	√	√
74	13.19_498.2257n	Angeloylgomisin O^*^	M+Na, 2M+Na, M+NH_4_	C_28_H_34_O_8_	0.70	√	√	√	√
75	14.15_514.2802*m/z*	Kadlongilactone F	M+NH_4_	C_29_H_36_O_7_	0.63				√
76	14.15_653.3302*m/z*	Gelsenicine	2M+H	C_19_H_22_N_2_O_3_	−4.87	√	√	√	√

* Indicates that it is identified by the standard.

### Evaluation of antioxidant activities

**Figure 6:**
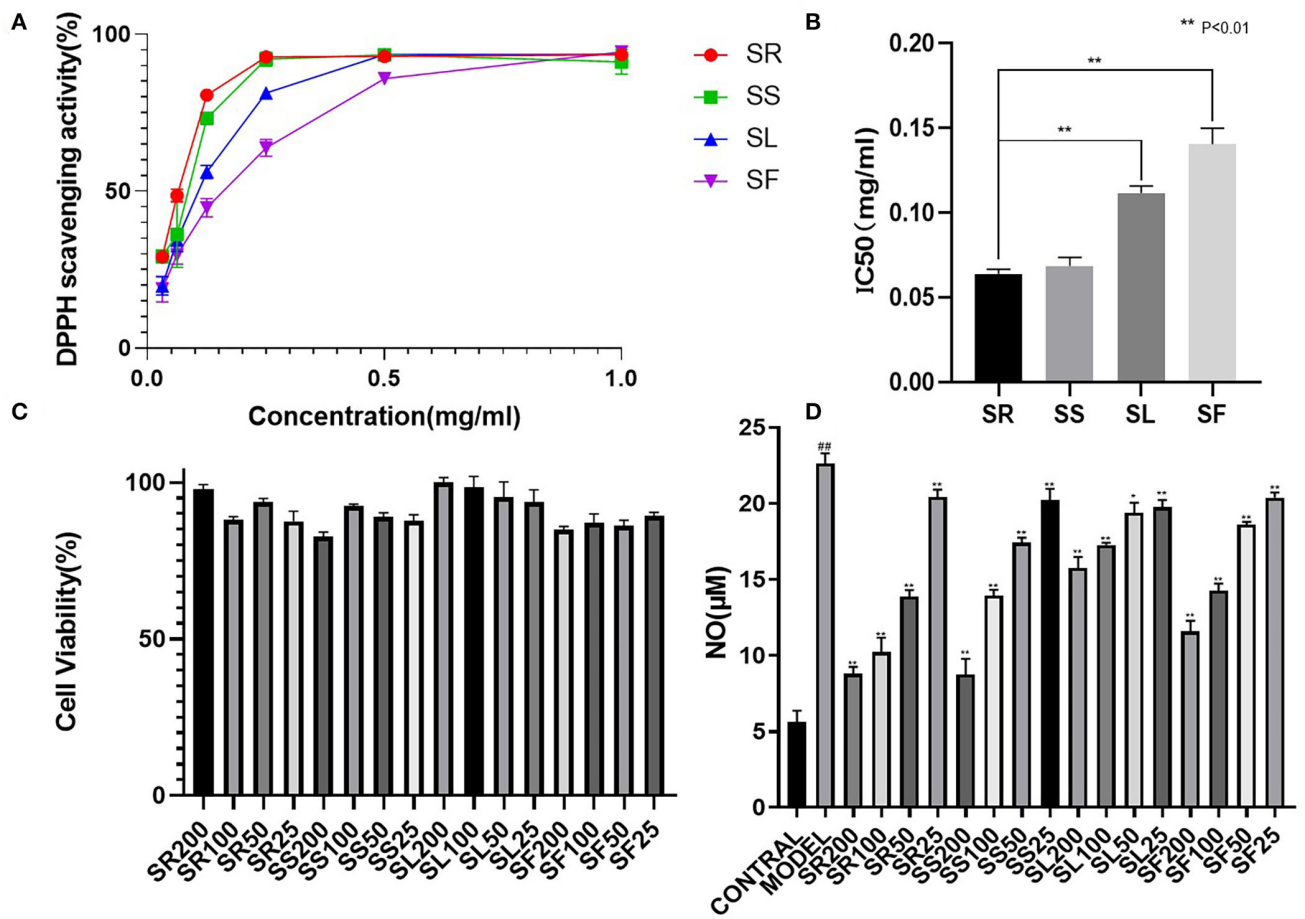
Comparative 2,2′-diphenyl-1-picrylhydrazyl (DPPH) scavenging capacity and anti-inflammatory activity of *S. chinensis*. **(A)** DPPH scavenging activity of different plant parts. **(B)** The half maximal inhibitory concentration (IC_50_) values of different plant parts. **(C)** Cell viability of different plant parts. **(D)** Anti-inflammatory activity of different plant parts. The results were expressed as the mean ± standard deviation, and triplicate experiments were performed for each sample.

2,2′-Diphenyl-1-picrylhydrazyl (DPPH) free radicals are stable nitrogen center free radicals, and their ethanolic solution is purple and has the largest absorption peak at 517 nm. With the addition of antioxidants, DPPH captures an electron and pairs with free electrons, and the purple fades and becomes colorless. Based on this principle, the antioxidant ability can be detected and is widely used in the research of antioxidant foods, health products, and drugs. Figure 6A shows the free radical scavenging activity of the SR, SS, SL, and SF from *S. chinensis* at different concentrations. 2,2′-Diphenyl-1-picrylhydrazyl radicals exhibited a dose-dependent effect; at concentrations above 0.5 mg/mL, the antioxidant activity showed no distinction. However, at low concentrations (0.031 mg/mL), the antioxidant activity of roots and stems was much higher than that of leaves and fruits. The IC_50_ (half maximal inhibitory concentration) is used as an indicator of antioxidant activities. Figure 6B shows that SR had the strongest antioxidant capacity (IC_50_ 0.064 ± 0.005 mg/mL), followed by SS (IC_50_ 0.069 ± 0.006 mg/mL), SL (IC_50_ 0.112 ± 0.007 mg/mL), and SF (IC_50_ 0.140 ± 0.013 mg/mL). One-way ANOVA revealed significant differences between the four groups (*p* < 0. 01). Overall, all the extracts from different parts of 95% ethanol exhibited DPPH scavenging activity, with the root activity being the highest (Figure 6).

### Evaluation of anti-inflammatory activity on RWA264.7 cells

The anti-inflammatory properties of the alcohol extracts from the selected medicinal herbs were evaluated based on their ability to inhibit the production of NO in LPS-activated mouse macrophages. The effects of the different parts on cell viability were evaluated by the CCK-8 assay.

The results of the cytotoxicity test showed that, below 200 μg/mL, the SR, SS, SL, and SF of *S. chinensis* were not cytotoxic; therefore, 200, 100, 50, and 25 μg/mL were selected to verify the anti-inflammatory activity. The NO content results showed that there was a very significant difference between the model and the blank (##, *P* < 0.01), suggesting that LPS (1 μg/mL) could induce an increase in NO produced by RAW264.7 cells, and 100 and 200 μg/mL of alcohol extracts of roots, stems, leaves, and fruits could significantly reduce the expression of NO (^**^, *P* < 0.01). Below 100 μg/mL, the effects of alcohol extracts from four different parts were SR > SS > SF > SL (Figure 6).

### Potential biomarkers from the root extracts

**Figure 7:**
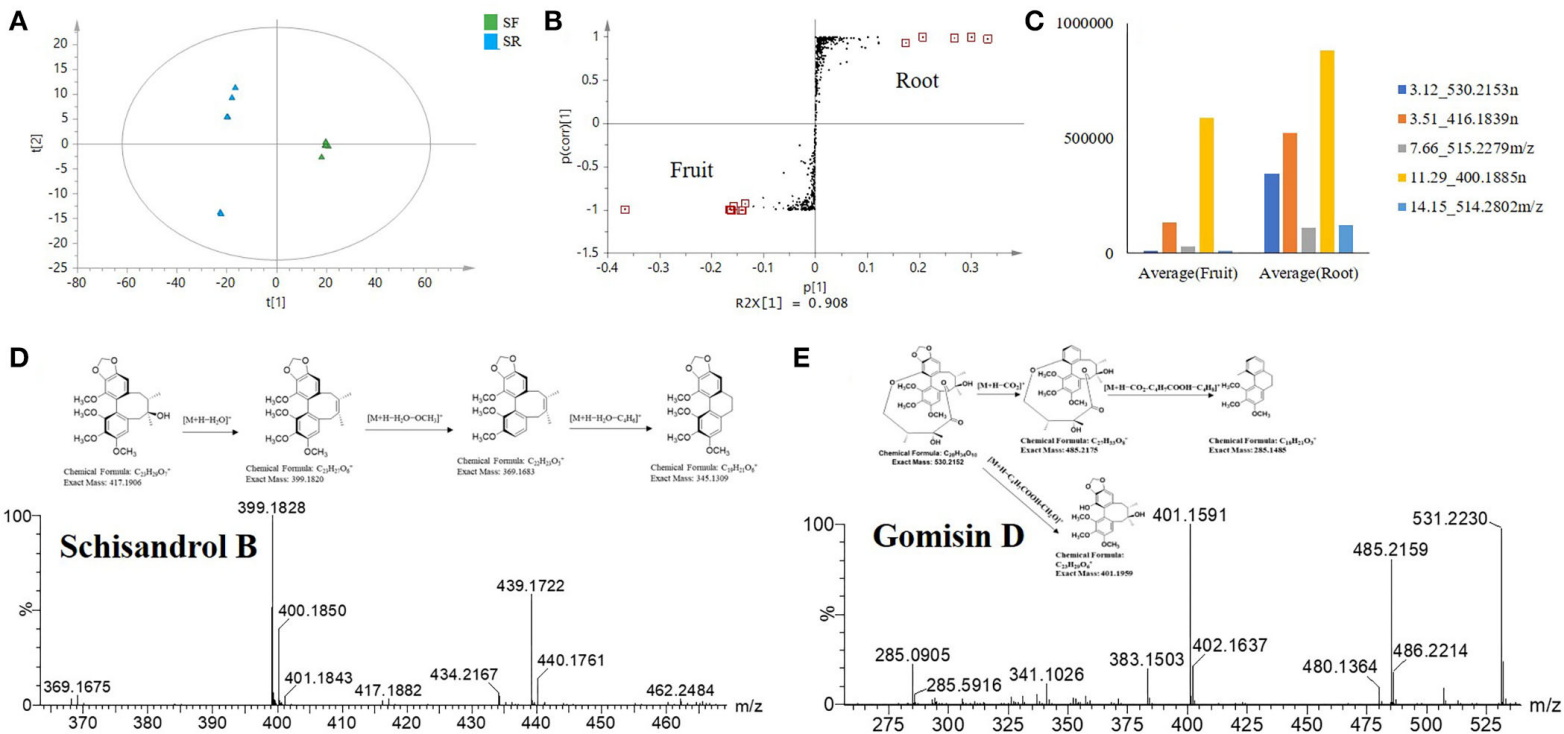
Selected *S. chinensis* potential biomarkers from the comparative analysis of SR and SF based on the S-plot. **(A)** OPLS-DA. **(B)** S-plot showing the selected markers. **(C)** Variable averages by a group of selected potential marker compounds. **(D)** Cracking process of schisandrol B. **(E)** Cracking process of gomisin D.

Recently, research on the antioxidant and anti-inflammatory activities of *S. chinensis* has mainly focused on SF extract. In our study, other parts of *S. chinensis* also showed significant antioxidant and anti-inflammatory activities. In addition, SR extracts exhibited higher antioxidant and anti-inflammatory activities than SF extracts. The chemical compositions of SR and SF were significantly different according to the PCA score plot (Figure 2). The potential biomarkers were identified by OPLS-DA together with an S-plot based on the difference shown in the PCA score plot (Figure 7).

The OPLS-DA statistical results showed the difference between SR and SF. The statistical model verified that the fit was good, with a cumulative R2 Y value of 0.99 and a Q2 value of 0.99 (Figure 7A). Twelve potential biomarkers were selected through the S-plot analysis of the SR and SF (Figure 7B). From the VIP plot analysis, the 12 selected potential biomarkers in SR had a higher VIP value (VIP > 3), indicating that these marker compounds were significantly different between SR and SF. In addition, the mean values of variables in each group showed that the selected compounds in SR and SF had different levels (Figure 7C). As shown in Figures 7B–D and Table 2, the top five potential biomarkers between SR and SF were 530.2153n (RT 3.12 min), 416.1839n (RT 3.51 min), 515.2279n (RT 7.66 min), 400.1885n (RT 11.29 min), and 514.2802 *m/z* (RT 14.15 min). The related compounds may play a key role in the higher biological activity of SR relative to SF.

The ion at 416.1839 *m/z* (RT 3.51 min) was identified as schisandrol B. The compound identification was based on the major matched fragment ions, including *m/z* 439.1736 [M+Na]^+^; in the process of substituent cracking with schisandrol B, H_2_O molecules were lost, and the fragment ions of [M+H-H_2_O]^+^ (*m/z* 399.1820) were found. Fragment ions including [M+H-H_2_O-OCH_3_]^+^ (*m/z* 368.1626) and [M+H_2_O-C_4_H_6_]^+^ (*m/z* 345.13) were found *via* further skeleton cracking reported in *S. chinensis* and were confirmed by reference standards (Figure 7D). The ion 530.2153n (RT 3.12 min) was identified as gomisin D, and the formula was C_24_H_32_O_6_. In the positive ion mode, fragment ions at *m/z* 531 [M+H]^+^, *m/z* 553 [M+Na]^+^, *m/z* 569 [M+K]^+^, *m/z* 485 [M+H-CO_2_]^+^, *m/z* 285 [M+H-CO_2_-C_4_H_7_COOH-C_4_H_8_]^+^, and *m/z* 401 [M+H-C_4_H_7_COOH-CH_2_O]^+^ were obtained. The main fragment ions produced were at *m/z* 485.2156 due to a loss of CO_2_ (44 Da), at *m/z* 401.1588 due to a further loss of C_4_H_7_COOH (100 Da) and CH_2_O (30 Da), and at *m/z* 285.0888 due to a loss of C_4_H_7_COOH (100 Da) and C_4_H_8_ (56 Da). The parent ions and the main fragmentation were consistent with the literature MS data for gomisin D (Figure 7E).

We detected the contents of 9 common lignans including schisandrol A (1), gomisin D (2), gomisin J (3), schisandrol B (4), angeloylgomisin H (5), gomisin G (6), schisantherin A (7), schisandrin B (8), and schisandrin C (8) in different parts of *S. chinensis*. The results indicated that the content of nine lignans varied greatly from different parts, and the total content of nine lignans was the highest in roots and the lowest in leaves. The content of schisandrol A and gomisin J in SF was higher than that in other parts. The content of gomisin D, schisandrol B, and schisantherin C in SR was significantly higher than that in other parts (Table 3).

**Table 3:** Contents of nine lignans in different parts of *Schisandra chinensis* (*n* = 3, mg/g).

		**1**	**2**	**3**	**4**	**5**	**6**	**7**	**8**	**9**	**Total**
S1	Fruit (SF)	12.79	1.08	2.13	5.31	2.40	0.57	0.54	7.22	1.35	33.39
	Stems (SS)	6.44	1.89	0.04	8.92	1.98	0.86	0.12	1.91	0.83	22.99
	Leaves (SL)	4.19	1.49	0.10	4.89	1.15	0.00	0.09	1.06	0.09	13.06
	Roots (SR)	6.79	24.13	0.02	12.97	2.09	0.67	0.49	10.34	0.96	58.46
S2	Fruit (SF)	11.09	1.12	1.72	4.80	2.01	0.58	0.36	8.87	2.27	32.82
	Stems (SS)	5.55	1.90	0.01	7.98	1.66	0.80	0.08	2.33	1.33	21.64
	Leaves (SL)	3.64	1.53	0.05	4.66	1.00	0.03	0.06	1.22	0.14	12.35
	Roots (SR)	5.59	23.27	0.00	11.29	1.72	0.64	0.31	12.20	1.46	56.48
S3	Fruit (SF)	12.72	0.78	1.27	3.29	1.90	0.33	0.22	6.34	1.69	28.54
	Stems (SS)	6.26	1.34	0.00	5.41	1.53	0.54	0.05	1.64	1.01	17.77
	Leaves (SL)	4.25	1.08	0.04	2.06	1.01	0.02	0.06	2.43	0.59	11.54
	Roots (SR)	6.74	17.16	0.00	8.10	1.68	0.43	0.22	9.71	1.00	45.05
S4	Fruit (SF)	13.81	1.13	1.59	3.80	2.38	0.52	0.43	7.24	1.37	32.27
	Stems (SS)	6.97	1.88	0.01	6.41	2.01	0.80	0.09	1.89	0.88	20.95
	Leaves (SL)	4.76	1.58	0.03	2.48	1.36	0.02	0.13	2.83	0.52	13.72
	Roots (SR)	7.64	25.36	0.00	9.65	2.19	0.65	0.44	11.47	0.89	58.29
S5	Fruit (SF)	12.35	0.91	1.61	2.98	1.90	0.60	0.32	5.51	1.05	27.23
	Stems (SS)	6.31	1.54	0.01	5.03	1.60	0.97	0.07	1.47	0.69	17.68
	Leaves (SL)	6.57	0.59	0.02	3.18	1.31	0.50	0.12	2.51	0.53	15.34
	Roots (SR)	6.48	22.74	0.00	7.00	1.66	0.42	0.29	8.38	0.62	47.58
S6	Fruit (SF)	10.08	1.08	1.05	2.67	1.67	0.51	0.35	4.67	0.85	22.93
	Stems (SS)	5.09	1.83	0.01	4.40	1.41	0.87	0.08	1.22	0.52	15.44
	Leaves (SL)	5.44	0.72	0.02	2.90	1.21	0.52	0.13	2.16	0.46	13.56
	Roots (SR)	5.35	27.56	0.00	6.36	1.50	0.32	0.32	7.32	0.54	49.25

Gomisin D is a lignan found in SFs and has been demonstrated to exert an antidiabetic effect and inhibit UDP-glucuronosyltransferase activity (Zhang et al., 2010; Song et al., 2015). In addition, gomisin D can scavenge ABTS (+) radicals and treat Alzheimer's disease (Mocan et al., 2016). Recently, gomisin D was used as a quality marker of Shengmai San and Shenqi Jiangtang Granule (Zhang et al., 2018). Schisandrol B is a lignan that can be isolated from dried SFs and exhibits hepatoprotective, cardioprotective, renoprotective, and memory-enhancing properties (Kim et al., 2008; Panossian and Wikman, 2008; Jiang et al., 2015, 2016; Zeng et al., 2017). There is also prior evidence that schisandrol B can partially suppress or prevent vascular fibrotic disorders by disrupting TGFβ1-assisted NF-κB signaling (Chun et al., 2018). In mice, schisandrol B can also drive benign liver enlargement, which is consistent with enhanced hepatocyte energy metabolism and energy utilization (Zhao et al., 2020). Kadsuranin, which is an anti-inflammatory marker that was identified, is also dibenzocyclooctene lignan derivative, and further experimental study may need to be performed to obtain the structure-activity relationship. Other researchers, who studied the antioxidant and anti-inflammatory activities of SF extracts, found that galloylated compounds, mainly dibenzocyclooctene lignan, are important bioactive constituents. However, gomisin D can be used as an important chemical marker of SR, which has significantly different contents in SF and SR. Gomisin D, schisandrol B, schisantherin C, kadsuranin, and kadlongilactone F could be considered chemical markers in the roots, which could support the root extracts has higher antioxidant and anti-inflammatory activities, and provide a new application direction for studying the parts with the antioxidant and anti-inflammatory activities from *S. chinensis*.

## Conclusion

A local compounds database from the genus *Schisandra* was established by Progenesis SDF Studio. The metabolite characterization in the root, stem, leaf and fruit of *Schisandra chinensis* was performed by a UPLC-QTOF-MS method using chemometrics tools to identify biomarkers of different parts of the plant. Through the screening of the antioxidant and anti-inflammatory activities of different parts of *S. chinensis in vitro*, we found alternative sources of antioxidants and anti-inflammatory compounds to study the correlation between chemical composition and biological activities of the plant parts of *S. chinensis*. Gomisin D, schisandrol B, schisantherin C, kadsuranin, and kadlongilactone F, as biomarkers from roots could support the material basis for the higher antioxidant and anti-inflammatory activities that are found in root extracts compared to fruits and provide a new application direction for studying the parts that exhibit the antioxidant and anti-inflammatory activities from *S. chinensis*. The plant roots could be a new medicinal source that exhibits better activity than that of traditional medicinal parts, which makes them worth exploring. In this study, the metabolic profiling of different parts of *S. chinensis* was characterized by using the theory of pharmacophylogeny, to expand the search for alternative drug sources. This study explored the chemical components and biological activities of the nonmedicinal parts of *S. chinensis*, which can be used to maximize the comprehensive utilization benefits of *S. chinensis* resources. Our next step is to isolate the labeled compounds o*f S. chinensis* and further confirm the biological activity of the compound.

## Credit authority statement

The work described in the manuscript has not been submitted elsewhere for publication as a whole. The relevant contents and data of the paper met the requirements of integrity. All authors have contributed and read the manuscript and agreed to its submission.

## Data availability statement

The original contributions presented in the study are included in the article/Supplementary material, further inquiries can be directed to the corresponding author/s.

## Author contributions

JiuL was responsible for data analysis and writing of the manuscript. XM, JinL, JZ, and TQ did experimental work. BL performed data analysis work. BZ and HoL collected and identified samples. HaL conceived the design of the study. All authors contributed to the article and approved the submitted version.

## Funding

The authors gratefully acknowledge the financial support by the National Natural Science Foundation of China (82204576), CAMS Innovation Fund for Medical Sciences (2021-I2M-1-031), and the Ability Establishment of Sustainable Use for Valuable Chinese Medicine Resources (2060302-2002-05).

## Conflict of interest

The authors declare that the research was conducted in the absence of any commercial or financial relationships that could be construed as a potential conflict of interest.

## Publisher's note

All claims expressed in this article are solely those of the authors and do not necessarily represent those of their affiliated organizations, or those of the publisher, the editors and the reviewers. Any product that may be evaluated in this article, or claim that may be made by its manufacturer, is not guaranteed or endorsed by the publisher.

## References

[B1] Brereton R. G. (2009). Chemometrics for Pattern Recognition. Hoboken, NJ: John Wiley and Sons.

[B2] Chaleckis R. Meister I. Zhang P. Wheelock C. E. (2019). Challenges, progress and promises of metabolite annotation for LC-MS-based metabolomics. Curr. Opin. Biotechnol. 55, 44–50. 10.1016/j.copbio.2018.07.010 30138778

[B3] Chen L. J. Hu P. P. Xue W. L. (2011). Research on tea beverages form fresh twigs and leaves of *Schisandra Chinense*. J. Anhui Agri. Sci. 39, 2647–2678. 10.13989/j.cnki.0517-6611.2011.05.004

[B4] Chen Q. Bao L. Lv L. Xie F. Zhou X. Zhang H.. (2021). Schisandrin B regulates macrophage polarization and alleviates liver fibrosis via activation of PPARγ. Ann. Transl. Med. 9, 1500. 10.21037/atm-21-4602 34805362 PMC8573433

[B5] Chen X. Tang R. Liu T. Dai W. Liu Q. Gong G.. (2019). Physicochemical properties, antioxidant activity and immunological effects *in vitro* of polysaccharides from *Schisandra sphenanthera* and *Schisandra chinensis*. Int. J. Biol. Macromol. 131, 744–751. 10.1016/j.ijbiomac.2019.03.129 30904534

[B6] Chinese Pharmacopoeia Commission (2020). The Pharmacopoeia of the People's Republic of China, 2020 Edition Part I. Beijing: China Medical Science Press.

[B7] Chun J. N. Park S. Lee S. Kim J. K. Park E. J. Kang M. J.. (2018). Schisandrol B and schisandrin B inhibit TGFβ1-mediated NF-κB activation via a Smad-independent mechanism. Oncotarget 9, 3121–3130. 10.18632/oncotarget.23213 29423034 PMC5790451

[B8] Dai W. D. Qi D. D. Yang T. Lv H. P. Guo L. Zhang Y.. (2015). Nontargeted analysis using ultraperformance liquid chromatography-quadrupole time-of-flight mass spectrometry uncovers the effects of harvest season on the metabolites and taste quality of tea (*Camellia sinensis* L.). J. Agric. Food Chem. 63, 9869–9878. 10.1021/acs.jafc.5b03967 26494158

[B9] Gui Y. Yang Y. Xu D. Tao S. Li J. (2020). Schisantherin A attenuates sepsis-induced acute kidney injury by suppressing inflammation via regulating the NRF2 pathway. Life Sci. 258, 118161. 10.1016/j.lfs.2020.118161 32730835

[B10] Guo L. Y. Hung T. M. Bae K. H. Shin E. M. Zhou H. Y. Hong Y. N.. (2008). Anti-inflammatory effects of schisandrin isolated from the fruit of *Schisandra chinensis* Baill. Eur. J. Pharmacol. 591, 293–299. 10.1016/j.ejphar.2008.06.074 18625216

[B11] Han J. S. Lee S. Kim H. Y. Lee C. H. (2015). MS-based metabolite profiling of aboveground and root components of *Zingiber mioga* and *officinale*. Molecules 20, 16170–16185. 10.3390/molecules200916170 26404226 PMC6332244

[B12] Hu D. Cao Y. He R. Han N. Liu Z. Miao L.. (2012). Schizandrin, an antioxidant lignan from *Schisandra chinensis*, ameliorates Aβ1-42-induced memory impairment in mice. Oxid. Med. Cell. Longev. 2012, 721721. 10.1155/2012/721721 22829961 PMC3399599

[B13] Hur M. Campbell A. A. Almeida-de-Macedo M. Li L. Ransom N. Jose A.. (2013). A global approach to analysis and interpretation of metabolic data for plant natural product discovery. Nat. Prod. Rep. 30, 565–583. 10.1039/c3np20111b 23447050 PMC3629923

[B14] Jandric Z. Frew R. D. Fernandez-Cedi L. N. Cannavan A. (2017). An investigative study on discrimination of honey of various floral and geographical origins using UPLC-QTOF-MS and multivariate data analysis. Food Control 72, 189–197. 10.1016/j.foodcont.2015.10.010

[B15] Jiang Y. M. Fan X. M. Wang Y. Chen P. Zeng H. Tan H.. (2015). Schisandrol B protects against acetaminophen-induced hepatotoxicity by inhibition of CYP-mediated bioactivation and regulation of liver regeneration. Toxicol. Sci. 143, 107–115. 10.1093/toxsci/kfu216 25319358 PMC4334815

[B16] Jiang Y. M. Wang Y. Tan H. S. Yu T. Fan X. M. Chen P.. (2016). Schisandrol B protects against acetaminophen-induced acute hepatotoxicity in mice via activation of the NRF2/ARE signaling pathway. Acta Pharmacol. Sin. 37, 382–389. 10.1038/aps.2015.120 26806302 PMC4775844

[B17] Kim S. H. Kim Y. S. Kang S. S. Bae K. Hung T. M. Lee S. M. (2008). Anti-apoptotic and hepatoprotective effects of gomisin A on fulminant hepatic failure induced by D-galactosamine and lipopolysaccharide in mice. J. Pharmacol. Sci. 106, 225–233. 10.1254/jphs.fp0071738 18270473

[B18] Lever J. Krzywinski M. Altman N. (2017). Points of significance: principal component analysis. Nat. Methods 14, 641–643. 10.1038/nmeth.4346

[B19] Lin C. C. Xu Z. Y. Wang B. H. Zhuang W. Y. Sun J. H. Li H.. (2021). Relaxation effect of *Schisandra chinensis* lignans on the isolated tracheal smooth muscle in rats and its mechanism. J. Med. Food 24, 825–832. 10.1089/jmf.2021.K.0037 34406878 PMC8403203

[B20] Liu G. Z. Liu Y. Sun Y. P. Li X. M. Xu Z. P. Jiang P.. (2020). Lignans and terpenoids from the leaves of *Schisandra chinensis*. Chem. Biodivers. 17, e2000035. 10.1002/cbdv.202000035 32141193

[B21] Lu B. Y. Li M. Q. Yin R. (2016). Phytochemical content, health benefits, and toxicology of common edible flowers: a review (2000–2015). Crit. Rev. Food Sci. Nutr. 56, 130–148. 10.1080/10408398.2015.1078276 26462418

[B22] Medica Editorial Board of National Institute of Chinese Medicine (1999). Chinese Materia Medica. Shanghai: Shanghai Scientific and Technical Publishers.

[B23] Mocan A. Schafberg M. Cri?an G. Rohn S. (2016). Determination of lignans and phenolic components of *Schisandra chinensis* (Turcz.) Baill. using HPLC-ESI-ToF-MS and HPLC-online TEAC: contribution of individual components to overall antioxidant activity and comparison with traditional antioxidant assays. J. Funct. Foods 24, 579–594. 10.1016/j.jff.2016.05.007

[B24] Mu X. L. Liu J. S. Li B. Wei X. P. Qi Y. D Zhang B. G.. (2022). A comparative study on chemical characteristics, antioxidant and hepatoprotective activity from different parts of *Schisandrae chinensis* Fructus. J. Food Process. Pres. 2022, e16990. 10.1111/jfpp.16990

[B25] Panossian A. Wikman G. (2008). Pharmacology of *Schisandra chinensis* Bail: an overview of Russian research and uses in medicine. J. Ethnopharmacol. 118, 183–212. 10.1016/j.jep.2008.04.020 18515024

[B26] Song J. H. Cui L. An L. B. Li W. T. Fang Z. Z. Zhang Y. Y.. (2015). Inhibition of UDP-glucuronosyltransferases (UGTS) activity by constituents of *Schisandra chinensis*. Phytother. Res. 29, 1658–1664. 10.1002/ptr.5395 26084208 PMC6594156

[B27] Szopa A. Barnaś M. Ekiert H. (2019). Phytochemical studies and biological activity of three Chinese Schisandra species (*Schisandra sphenanthera, Schisandra henryi* and *Schisandra rubriflora*): current findings and future applications. Phytochem. Rev. 18, 109–128. 10.1007/s11101-018-9582-0

[B28] Szopa A. Ekiert R. Ekiert H. (2017). Current knowledge of *Schisandra chinensis* (Turcz.) Baill. (Chinese magnolia vine) as a medicinal plant species: a review on the bioactive components, pharmacological properties, analytical and biotechnological studies. Phytochem. Rev. 16, 195–218. 10.1007/s11101-016-9470-4 28424569 PMC5378736

[B29] Trygg J. Holmes E. Lundstedt T. (2007). Chemometrics in metabonomics. J. Proteome Res. 6, 469–479. 10.1021/pr060594q 17269704

[B30] Usman M. Bokhari S. A. M. Fatima B. Rashid B. Nadeem F. Sarwar M. B. (2022). Drought stress mitigating morphological, physiological, biochemical, and molecular responses of guava (*Psidium guajava* L.) cultivars. Front. Plant Sci. 13:878616. 10.3389/fpls.2022.878616 35720611 PMC9201916

[B31] Wang X. Yu J. Li W. Wang C. Li H. Ju W.. (2018). Characteristics and antioxidant activity of lignans in *Schisandra chinensis* and *Schisandra sphenanthera* from different locations. Chem. Biodivers. 15, e1800030. 10.1002/cbdv.201800030 29706012

[B32] Xu G. Y. Lv X. Feng Y. B. Li H. Chen C. Lin H.. (2021). Study on the effect of active components of *Schisandra chinensis* on liver injury and its mechanisms in mice based on network pharmacology. Eur. J. Pharmacol. 910, 174442. 10.1016/j.ejphar.2021.174442 34492285

[B33] Xu J. B. Gao G. C. Yuan M. J. Huang X. Zhou H. Y. Zhang Y.. (2020). Lignans from *Schisandra chinensis* ameliorate alcohol and CCl_4_-induced long-term liver injury and reduce hepatocellular degeneration via blocking ETBR. J. Ethnopharmacol. 258, 112813. 10.1016/j.jep.2020.112813 32259665

[B34] Xu L. Grandi N. Del V. C. Mandas D. Corona A. Piano D.. (2015). From the traditional Chinese medicine plant *Schisandra chinensis* new scaffolds effective on HIV-1 reverse transcriptase resistant to non-nucleoside inhibitors. J. Microbiol. 53, 288–293. 10.1007/s12275-015-4652-0 25740376

[B35] Yan T. Wang N. Liu B. Wu B. Xiao F. He B.. (2021). *Schisandra chinensis* ameliorates depressive-like behaviors by regulating microbiota-gut-brain axis via its anti-inflammation activity. Phytother. Res. 35, 289–296. 10.1002/ptr.6799 32761702

[B36] Yang K. Qiu J.ing. Huang Z. C. Yu Z. W. Wang W. J. Hu H. L.. (2021). A comprehensive review of ethnopharmacology, phytochemistry, pharmacology, and pharmacokinetics of *Schisandra chinensis* (Turcz.) Baill. and *Schisandra sphenanthera* Rehd. et Wils. J. Ethnopharmacol. 284, 114759. 10.1016/j.jep.2021.114759 34678416

[B37] Yang S. Yuan C. H. (2021). *Schisandra chinensis*: a comprehensive review on its phytochemicals and biological activities. Arab. J. Chem. 14, 103310. 10.1016/j.arabjc.2021.10331034678416

[B38] Ye J. W. Zhang Z. K. Wang H. F. Bao L. Ge J. P. (2019). Phylogeography of *Schisandra chinensis* (Magnoliaceae) reveal multiple refugia with ample gene flow in Northeast China. Front. Plant Sci. 10:199. 10.3389/fpls.2019.00199 30858859 PMC6397880

[B39] Zeng H. Jiang Y. M. Chen P. Li D. S. Li D. S. Liu D. S.. (2017). Schisandrol B protects against cholestatic liver injury through pregnane X receptors. Brit. J. Pharmacol. 174, 672–688. 10.1111/bph.13729 28128437 PMC5368048

[B40] Zhang H. Zhang X. J. Jiang H. J. Xu C. Tong S. Q. Yan J. Z. (2018). Screening and identification of alpha-glucosidase inhibitors from shenqi jiangtang granule by ultrafiltration liquid chromatography and mass spectrometry. J. Sep. Sci. 41, 797–805. 10.1002/jssc.201700835 29152897

[B41] Zhang J. Shi L. L. Zheng Y. N. (2010). Dibenzocyclooctadiene lignans from fructus *Schisandrae chinensis* improve glucose uptake *in vitro*. Nat. Prod. Commun. 5, 231–234. 10.1177/1934578X1000500212 20334133

[B42] Zhao Y. Y. Yao X. P. Jiao T. Y. Tian J. N. Zhou Y. Y. Gao Y.. (2020). Lipidomics analysis on schisandrol B-induced liver enlargement in mice. Acta Pharm. Sin. 12, 922–929. 10.16438/j.0513-4870.2019-1020

