# Supplementary material for: Metabolic profiling on the analysis of different parts of Schisandra chinensis based on UPLC-QTOF-MS with comparative bioactivity assays
Source: Front Plant Sci. 2022 Nov 28;13:970535. doi: 10.3389/fpls.2022.970535 (PMC9742558; doi:10.3389/fpls.2022.970535)
Supplement: Supplementary file 1 [file Data_Sheet_1.docx]

Table S1. A total of 332 metabolites identified

| no. | Compound | Adducts | Formula | Score | Mass Error (ppm) | Isotope Similarity | Description |
| --- | --- | --- | --- | --- | --- | --- | --- |
| 1 | 1.04_1097.2350m/z | 2M+H | C_25_H_24_O_14_ | 28 | -5.01 | 45.42 | Flavone base + 3O, 1MeO, O-MalonylHex |
| 2 | 1.04_291.0874m/z | M+H | C_15_H_14_O_6_ | 33.6 | 3.61 | 66.17 | (+)-Epicatechin |
| 3 | 1.04_371.1627m/z | M+NH4 | C_20_H_19_NO_5_ | 34.8 | 7.11 | 81.08 | (+)-Chelidonine |
| 4 | 1.04_379.0391m/z | 2M+Na | C_9_H_6_O_4_ | 35.5 | -9.32 | 86.99 | 7,8-Dihydroxycoumarin |
| 5 | 1.04_437.2049m/z | M+Na | C_23_H_30_N_2_O_5_ | 38.3 | 0.41 | 91.85 | 7-Hydroxymitragynine |
| 6 | 1.04_448.1017n | M+Na, 2M+Na, M+H | C_21_H_20_O_11_ | 40.4 | 2.56 | 84.68 | Plantaginin |
| 7 | 1.04_464.0911n | M+Na, 2M+Na, M+H | C_21_H_20_O_12_ | 41.1 | -9.42 | 92.08 | Quercetin-3-O-glucoside |
| 8 | 1.04_469.1488m/z | M+Na | C_23_H_26_O_9_ | 35.7 | 4.3 | 83.61 | Tiegusanin M |
| 9 | 1.04_579.1526m/z | M+H | C_30_H_26_O_12_ | 37.1 | 5.04 | 90.06 | Procyanidin B2 |
| 10 | 1.04_599.1170m/z | 2M+Na | C_15_H_12_O_6_ | 37.4 | 1.69 | 88.88 | Dihydrokaempferol |
| 11 | 1.04_617.1458m/z | M+Na | C_27_H_30_O_15_ | 36.5 | -3.12 | 85.83 | Vicenin 2 |
| 12 | 1.04_849.6459m/z | 2M+Na | C_27_H_43_NO_2_ | 28.5 | -2.53 | 45.48 | Petiline |
| 13 | 1.11_1119.4461m/z | 2M+Na | C_28_H_36_O_11_ | 32.1 | 4.88 | 66.32 | Xuetongdilactone F |
| 14 | 1.11_509.2170m/z | M+H | C_29_H_32_O_8_ | 36.7 | 0.02 | 83.48 | Longipedlignan K |
| 15 | 1.11_542.2133n | M+H, 2M+Na | C_29_H_34_O_10_ | 36.7 | -3.48 | 87.48 | Schindilactone H |
| 16 | 1.11_544.2274n | M+Na, 2M+Na, M+NH4 | C_29_H_36_O_10_ | 34.1 | -6.25 | 77.7 | Schindilactone C |
| 17 | 1.18_146.0606m/z | M+H | C_9_H_7_NO | 37.9 | 4.19 | 92.19 | 4-Hydroxyquinoline |
| 18 | 1.21_574.2210n | M+Na, 2M+Na | C_28_H_34_N_2_O_11_ | 36.2 | 8.31 | 90.08 | 5(S)-5-carboxystrictosidine |
| 19 | 1.21_599.2824m/z | 2M+Na | C_16_H_20_N_2_O_3_ | 36.8 | -2.78 | 87.45 | Indole-3-acetyl-L-isoleucine |
| 20 | 1.28_516.2675n | M+Na, 2M+Na, M+NH4 | C_29_H_40_O_8_ | 32.6 | -9.38 | 73.14 | Wuweizidilactones D |
| 21 | 1.31_287.0563m/z | M+H | C_15_H_10_O_6_ | 38.7 | 4.58 | 98.59 | Luteolin |
| 22 | 1.45_679.3075m/z | 2M+H | C_20_H_21_NO_4_ | 35.8 | 8.95 | 88.72 | Nantenine |
| 23 | 1.53_441.1875m/z | M+Na | C_23_H_30_O_7_ | 38.6 | -1.97 | 95.34 | Gomisin T |
| 24 | 1.60_819.4292m/z | 2M+Na | C_23_H_30_N_2_O_4_ | 35 | -1.43 | 76.53 | Mitragynine |
| 25 | 1.64_343.1524m/z | M+H | C_20_H_22_O_5_ | 35.5 | -4.56 | 82.56 | Chicanine |
| 26 | 1.64_375.2143m/z | M+H | C_22_H_30_O_5_ | 35.9 | -6.22 | 86.64 | Schineolignins B |
| 27 | 1.67_588.2571n | M+NH4, M+Na | C_31_H_40_O_11_ | 37.9 | 0.11 | 89.59 | pre-schisanartanin A |
| 28 | 1.67_627.2190m/z | M+Na | C_34_H_36_O_10_ | 37.6 | -1.83 | 90.4 | Longipedunin E |
| 29 | 1.70_328.1397m/z | M+H | C_15_H_21_NO_7_ | 38.8 | 1.79 | 95.54 | N-Fructosyl phenylalanine |
| 30 | 1.70_364.1524n | M+H, 2M+H | C_22_H_22_NO_4_ | 37.3 | -6.93 | 93.96 | Coralyne |
| 31 | 1.70_565.2842m/z | M+Na | C_30_H_42_N_2_O_7_ | 34 | -7.73 | 78.66 | Puberanidine |
| 32 | 1.81_603.3846m/z | 2M+H | C_15_H_27_NO_5_ | 38.6 | -0.85 | 94.08 | Trachelanthine |
| 33 | 1.84_657.2447m/z | 2M+Na | C_17_H_19_NO_5_ | 33.6 | 4.49 | 73.29 | Piperlongumine |
| 34 | 1.91_680.2899m/z | M+NH4 | C_33_H_42_O_14_ | 38.8 | -2.03 | 96.29 | Schisandroside D |
| 35 | 1.94_1107.4630m/z | 2M+H | C_30_H_35_NO_9_ | 32.7 | -5.96 | 70.32 | Nicotinoylgomisin Q |
| 36 | 1.97_240.2326m/z | M+NH4 | C_15_H_26_O | 37.1 | 1.91 | 87.89 | Isoepicubenol |
| 37 | 2.06_277.1780m/z | M+Na | C_15_H_26_O_3_ | 26.2 | 2.21 | 33.56 | 4-H-guaia-6-en |
| 38 | 2.06_574.2751n | M+NH4, M+Na | C_31_H_42_O_1_0 | 37.3 | -4.77 | 91.85 | Wuweizidilactones C |
| 39 | 2.10_635.2476m/z | M+Na | C_33_H_40_O_11_ | 36.7 | 2.14 | 85.9 | Wuweizidilactone A |
| 40 | 2.20_483.2023m/z | M+H | C_27_H_30_O_8_ | 38.2 | 2.06 | 93.36 | Isointeriorin |
| 41 | 2.23_530.2171n | M+NH4, M+Na, M+H | C_28_H_34_O_10_ | 33.1 | 3.67 | 69.76 | Taiwanschirin D |
| 42 | 2.30_526.2189n | M+NH4, M+Na, M+H | C_29_H_34_O_9_ | 38.3 | -2.54 | 94.3 | Schindilactone A |
| 43 | 2.33_261.1829m/z | M+Na | C_15_H_26_O_2_ | 39.1 | 1.48 | 97.06 | Alismoxide |
| 44 | 2.33_441.1865m/z | M+Na | C_23_H_30_O_7_ | 38.1 | -4.47 | 95.7 | (6S,7S,12aR)-5,6,7,8-Tetrahydro-2,3,10,11,12-pentamethoxy-6,7-Dimethyldibenzo[a,c]cyclooctene-1,7-diol |
| 45 | 2.43_581.2760m/z | M+Na | C_31_H_42_O_9_ | 36.4 | 7.03 | 89.86 | Wuweizidilactones E |
| 46 | 2.43_733.3994m/z | 2M+H | C_22_H_26_N_2_O_3_ | 37.8 | 4.65 | 93.51 | Hirsuteine |
| 47 | 2.47_709.2249m/z | 2M+H | C_20_H_18_O_6_ | 38.1 | -4.26 | 94.52 | Triacetyl resveratrol |
| 48 | 2.52_610.2420n | M+H, M+Na | C_33_H_38_O_11_ | 36.3 | 1.01 | 82.83 | Schizanrin H |
| 49 | 2.56_544.3628m/z | M+NH4 | C_32_H_46_O_6_ | 39.3 | -0.83 | 97.5 | Heteroclitalactone A |
| 50 | 2.56_572.3968m/z | M+NH4 | C_34_H_50_O_6_ | 33.3 | 4.07 | 71.41 | Heteroclitalactone C |
| 51 | 2.59_358.1775n | M+H, M+Na | C_21_H_26_O_5_ | 39 | -1.39 | 96.87 | Grandisin |
| 52 | 2.73_691.3236m/z | 2M+Na | C_21_H_22_N_2_O_2_ | 32.8 | -2.84 | 67.51 | Strychnine |
| 53 | 2.76_284.0696n | 2M+H, 2M+Na, M+H, M+Na | C_16_H_12_O_5_ | 39.9 | 3.99 | 84.09 | Wogonin |
| 54 | 2.76_353.1734m/z | M+Na | C_20_H_26_O_4_ | 38.3 | 3.17 | 95.28 | Schineolignins A |
| 55 | 2.76_432.2149n | M+H, M+NH4, 2M+Na | C_24_H_32_O_7_ | 37.4 | 0.14 | 87.12 | Schisandrol A* |
| 56 | 2.76_668.3034m/z | M+Na | C_34_H_47_NO_11_ | 39.1 | -1.1 | 96.67 | Aconitine |
| 57 | 2.76_709.2963m/z | 2M+H | C_21_H_22_O_5_ | 26.3 | -6.2 | 36.11 | Chalcone base + 3O, 1MeO, 1Prenyl |
| 58 | 2.76_743.2532m/z | 2M+Na | C_18_H_20_N_2_O_6_ | 33.2 | -0.44 | 62.91 | Isodityrosine |
| 59 | 2.79_656.2827n | M+NH4, M+Na, M+H | C_35_H_44_O_12_ | 37.3 | -0.94 | 87.55 | Wuweizidilactone B |
| 60 | 2.83_975.1847m/z | 2M+Na | C_22_H_20_O_12_ | 27.9 | 4.77 | 43.19 | Flavone base + 3O, 1MeO, O-HexA |
| 61 | 3.05_277.1773m/z | M+Na | C_15_H_26_O_3_ | 31.9 | -0.28 | 60.06 | 4-trihydroxy-1-H-guaia-6-en |
| 62 | 3.12_530.2153n | M+Na, 2M+Na, 2M+H, M+NH4, M+H | C_28_H_34_O_10_ | 38.5 | 0.12 | 92.59 | Gomisin D* |
| 63 | 3.12_605.1460m/z | M+Na | C_26_H_30_O_15_ | 29.5 | -2.86 | 49.79 | Flavanone base + 4O, O-Hex, C-Pen |
| 64 | 3.12_618.2893m/z | M+NH4 | C_32_H_40_O_11_ | 33.2 | -2.65 | 69.19 | Kadheterin H |
| 65 | 3.12_893.2518m/z | 2M+H | C_22_H_22_O_10_ | 35 | 2.2 | 76.89 | Flavone base + 2O, 1MeO, C-Hex |
| 66 | 3.15_539.2958m/z | M+H | C_32_H_42_O_7_ | 36.3 | -8.4 | 90.9 | Schinchinenlactone D |
| 67 | 3.15_590.2722n | M+NH4, M+Na | C_31_H_42_O_11_ | 38.2 | -0.82 | 92.22 | Pre-schisanartanin B |
| 68 | 3.22_259.1679m/z | M+Na | C_15_H_24_O_2_ | 38.1 | 4.5 | 95.54 | 4-dihydroxy-1-H-guaia-6,10(14)-dien |
| 69 | 3.22_497.2856m/z | 2M+H | C_15_H_20_O_3_ | 36.7 | -6.39 | 92.26 | Atractylenolide III |
| 70 | 3.25_545.1778m/z | M+Na | C_29_H_30_O_9_ | 38.6 | -0.75 | 93.77 | Longipedlignan B |
| 71 | 3.25_560.3561m/z | M+NH4 | C_32_H_46_O_7_ | 37.5 | -3.74 | 91.66 | Xuetongsu D |
| 72 | 3.29_389.1980m/z | M+H | C_22_H_28_O_6_ | 34.2 | -5.97 | 78.04 | Gomisin J* |
| 73 | 3.29_466.2420m/z | M+NH4 | C_24_H_32_O_8_ | 36.4 | -3.43 | 86.19 | Kadlongirins A |
| 74 | 3.29_580.1782n | M+NH4, M+Na | C_27_H_32_O_14_ | 33.8 | -1.66 | 70.17 | Naringin |
| 75 | 3.51_229.0880m/z | M+H | C_14_H_12_O_3_ | 39.8 | 9.28 | 91.81 | Cis-resveratrol |
| 76 | 3.51_254.0595n | M+H, 2M+H, M+NH4 | C_15_H_10_O_4_ | 35.3 | 6.37 | 80.67 | Daidzein |
| 77 | 3.51_295.1213m/z | M+Na | C_19_H_16_N_2_ | 36.1 | 2.86 | 83.33 | Sempervirine |
| 78 | 3.51_361.2008m/z | M+H | C_21_H_28_O_5_ | 37.8 | -0.39 | 89.25 | Kadangustin K |
| 79 | 3.51_387.1802m/z | M+H | C_22_H_26_O_6_ | 30.3 | -0.08 | 51.66 | Schisanhenol B |
| 80 | 3.51_404.1837n | M+H, M+Na, 2M+Na | C_22_H_28_O_7_ | 38.8 | 0.41 | 94.38 | Deangeloylschisantherin F |
| 81 | 3.51_416.1839n | M+Na, 2M+Na, M+NH4 | C_23_H_28_O_7_ | 38.2 | 0.98 | 92.36 | Schisandrol B* |
| 82 | 3.51_490.2796m/z | M+Na | C_25_H_41_NO_7_ | 35.5 | 4.5 | 82.74 | Lycoctonine |
| 83 | 3.51_546.2698m/z | M+NH4 | C_29_H_36_O_9_ | 38.5 | 0.14 | 92.79 | Schindilactone F |
| 84 | 3.51_573.2674m/z | M+Na | C_29_H_42_O_1_0 | 37.5 | 0.77 | 88.35 | Heteroclitalactone N |
| 85 | 3.51_781.2712m/z | 2M+H | C_20_H_22_O_8_ | 28.5 | 1.33 | 44.01 | 8,8'-dihydroxypinoresinol |
| 86 | 3.51_819.2605m/z | 2M+Na | C_22_H_22_O_7_ | 38.5 | -2.24 | 94.93 | Desoxypodophyllotoxin |
| 87 | 3.51_839.3827m/z | 2M+Na | C_25_H_28_O_5_ | 25.3 | 7.49 | 34.72 | Dihydroflavonol + 2O, 2Prenyl |
| 88 | 3.64_439.2307m/z | 2M+Na | C_11_H_16_N_2_O_2_ | 38.8 | -2.05 | 96.43 | Pilocarpine |
| 89 | 3.64_469.1843m/z | M+Na | C_24_H_30_O_8_ | 37.6 | 2.34 | 90.86 | Kadlongirins B |
| 90 | 3.64_587.1365m/z | M+Na | C_26_H_28_O_14_ | 32.8 | -1.16 | 64.95 | Flavone base + 3O, C-Pen-Hex |
| 91 | 3.64_681.3660m/z | 2M+H | C_20_H_24_N_2_O_3_ | 38 | 2.02 | 92.34 | Yohimbic Acid |
| 92 | 3.81_495.2453m/z | M+NH4 | C_28_H_31_NO_6_ | 35.4 | -7.58 | 85.24 | Speciosine |
| 93 | 3.92_514.3167m/z | M+NH4 | C_30_H_40_O_6_ | 37.3 | 0.81 | 87.26 | Longipedlactone C |
| 94 | 3.95_343.1522m/z | M+H | C_20_H_22_O_5_ | 37.4 | -5.22 | 93.01 | 4-[(2R,3S,4R,5R)-5-(1,3-Benzodioxol-5-yl)tetrahydro-3,4-dimethyl-2-furanyl]-2-methoxyphenol |
| 95 | 4.10_532.3249m/z | M+NH4 | C_30_H_42_O_7_ | 36.4 | -3.75 | 86.35 | Wuweizidilactone D |
| 96 | 4.14_523.2269m/z | M+Na | C_28_H_36_O_8_ | 38.3 | -6.67 | 99.09 | Tigloylgomisin H |
| 97 | 4.14_546.3060m/z | M+NH4 | C_30_H_40_O_8_ | 38.5 | -0.36 | 92.75 | Longipedlactone I |
| 98 | 4.17_465.2979m/z | M+H | C_30_H_40_O_4_ | 38.1 | -4.47 | 95.81 | Schisanlactone A |
| 99 | 4.31_1039.2277m/z | 2M+Na | C_23_H_24_O_13_ | 26.1 | -4.79 | 36.02 | Syringetin-3-O-glucoside |
| 100 | 4.31_1091.1887m/z | 2M+Na | C_24_H_22_O_14_ | 25.2 | -2.3 | 28.57 | Flavone base + 4O, O-MalonylHex |
| 101 | 4.31_390.2037n | M+H, M+Na | C_22_H_30_O_6_ | 39 | -1.44 | 96.77 | Pregomisin* |
| 102 | 4.31_917.3238m/z | 2M+H | C_24_H_26_O_9_ | 37.5 | 1.28 | 88.82 | Kadsutherin H |
| 103 | 4.44_453.1888m/z | M+Na | C_24_H_30_O_7_ | 38.3 | 1.03 | 92.87 | Kadsulignan N |
| 104 | 4.44_467.2044m/z | M+Na | C_25_H_32_O_7_ | 38.6 | 0.78 | 93.79 | Schisanhenol acetate |
| 105 | 4.53_282.2794m/z | M+NH4 | C_18_H_32_O | 36.2 | 0.93 | 81.9 | Guai-3-en-ol |
| 106 | 4.53_404.2416m/z | M+NH4 | C_23_H_30_O_5_ | 34 | -4.03 | 74.74 | (-)-Gomisin K1 |
| 107 | 4.56_401.1617m/z | M+H | C_22_H_24_O_7_ | 37.7 | 5.49 | 95.02 | Gomisin R |
| 108 | 4.56_518.2401m/z | M+NH4 | C_27_H_32_O_9_ | 37.4 | 3.22 | 90.65 | Longipedlignan E |
| 109 | 4.63_240.0872m/z | M+NH4 | C_11_H_10_O_5_ | 31.8 | 2.7 | 60.61 | Isofraxidin |
| 110 | 4.63_357.1353m/z | 2M+H | C_10_H_10_O_3_ | 31 | 5.8 | 60.77 | 3-Methoxycinnamic acid |
| 111 | 4.63_439.1728m/z | M+Na | C_23_H_28_O_7_ | 39.5 | 0.23 | 98.03 | 5,6,7,8-Tetrahydro-1,2,3,13-tetramethoxy-6,7-Dimethylbenzo[3,4]cycloocta[1,2-f][1,3]benzodioxol-5-ol |
| 112 | 4.63_552.1084n | M+H, M+Na | C_31_H_20_O_10_ | 37.5 | 5.04 | 93.04 | Biflavonoid-flavone base + 3O and flavone Base + 2O + 1MeO |
| 113 | 4.67_315.0873m/z | M+H | C_17_H_14_O_6_ | 36.9 | 3.23 | 87.09 | Flavone + 2O + 2MeO |
| 114 | 4.67_839.3723m/z | 2M+Na | C_25_H_28_O_5_ | 30.9 | -5.27 | 58.94 | Licodione base + 2Prenyl |
| 115 | 4.73_401.1961m/z | M+H | C_23_H_28_O_6_ | 39.6 | 0.54 | 98.75 | Kadsuranin |
| 116 | 4.73_518.2750m/z | M+NH4 | C_28_H_36_O_8_ | 39.2 | 0.32 | 96.28 | Heteroclitin A |
| 117 | 4.73_523.2308m/z | M+Na | C_28_H_36_O_8_ | 39.5 | 1.09 | 98.6 | Angeloylgomisin H* |
| 118 | 4.73_539.2040m/z | M+Na | C_26_H_32_N_2_O_9_ | 36 | 7.73 | 88.11 | Strictosidinic acid |
| 119 | 4.73_557.2031m/z | M+Na | C_27_H_34_O_11_ | 35.4 | 7.12 | 84.98 | Schisandroside C |
| 120 | 4.73_574.3353m/z | M+NH4 | C_32_H_44_O_8_ | 37.8 | -3.81 | 93.66 | Xuetongdilactone C |
| 121 | 4.73_578.1805n | M+H, M+NH4, 2M+Na, 2M+H | C_31_H_30_O_11_ | 35.1 | 2.84 | 78.98 | Kadsulignan G |
| 122 | 4.73_600.3523m/z | M+NH4 | C_34_H_46_O_8_ | 36.3 | -1.44 | 83.46 | Xuetonglactone F |
| 123 | 4.80_771.2624m/z | 2M+Na | C_20_H_22_O_7_ | 30.1 | 0.08 | 50.7 | 8-hydroxypinoresinol |
| 124 | 4.84_587.2129m/z | M+Na | C_28_H_36_O_12_ | 35.2 | 5.35 | 82.24 | Schisandroside A |
| 125 | 4.84_625.1757m/z | M+H | C_28_H_32_O_16_ | 37.3 | -1.05 | 87.38 | Flavone base + 3O, 1MeO, C-Hex-Hex |
| 126 | 4.94_302.1080m/z | M+NH4 | C_10_H_12_N_4_O_6_ | 22.6 | -5.46 | 18.07 | Xanthosine |
| 127 | 4.94_522.2256n | M+Na, 2M+Na, M+NH4 | C_30_H_34_O_8_ | 39.3 | 0.49 | 97.26 | Benzoylgomisin H |
| 128 | 5.02_343.1915m/z | M+H | C_21_H_26_O_4_ | 35.2 | 3.38 | 79.83 | Saururenin |
| 129 | 5.02_484.2108n | M+Na, 2M+Na, M+H | C_27_H_32_O_8_ | 39.1 | 2.25 | 98.38 | Angeloylbinankadsurin A |
| 130 | 5.13_372.2180m/z | M+NH4 | C_22_H_26_O_4_ | 36.5 | 3.05 | 86.14 | R(+) -gomisin M1 |
| 131 | 5.13_482.3032n | M+H, M+Na | C_30_H_42_O_5_ | 39.5 | -0.15 | 97.86 | Wuweizilactone acid |
| 132 | 5.16_619.2295m/z | 2M+Na | C_18_H_18_O_4_ | 38.4 | -1.18 | 93.15 | Enterolactone |
| 133 | 5.23_277.1785m/z | M+Na | C_15_H_26_O_3_ | 33.4 | 4.2 | 71.92 | Trihydroxy-H-guaia-6-en |
| 134 | 5.30_423.1884m/z | M+Na | C_22_H_28_N_2_O_5_ | 29.3 | -1.59 | 46.79 | Reserpic acid |
| 135 | 5.36_1183.4518m/z | 2M+Na | C_32_H_36_O_10_ | 34 | 0.78 | 70.85 | Kadheterin A |
| 136 | 5.36_206.0599n | M+Na, 2M+Na | C_11_H_10_O_4_ | 34.9 | 9.78 | 83.94 | Scoparone |
| 137 | 5.36_223.0743m/z | M+H | C_15_H_10_O_2_ | 39.1 | -4.86 | 86.53 | Flavone |
| 138 | 5.36_225.0905m/z | M+H | C_15_H_12_O_2_ | 35.3 | -2.26 | 72.79 | Flavanone |
| 139 | 5.36_341.1388m/z | M+H | C_20_H_20_O_5_ | 34.9 | 1.2 | 68.19 | Arylbenzofuran flavonoid base + 3O, 1MeO, 1Prenyl |
| 140 | 5.36_430.1996n | M+H, M+Na, 2M+H, 2M+Na | C_24_H_30_O_7_ | 38.8 | 0.96 | 95.19 | Schisanchinins C |
| 141 | 5.36_576.3173m/z | M+NH4 | C_31_H_42_O_9_ | 39.6 | 1.09 | 99.12 | Propindilactone J |
| 142 | 5.36_592.3116m/z | M+NH4 | C_31_H_42_O_10_ | 37.2 | -0.03 | 86.28 | Wuweizidilactones F |
| 143 | 5.36_943.5224m/z | M+H | C_48_H_78_O_1_8 | 34.3 | -3.93 | 76.01 | Soyasaponin Bb |
| 144 | 5.40_659.3323m/z | 2M+H | C_19_H_23_NO_4_ | 29.4 | -0.59 | 47.66 | Sinomenine |
| 145 | 5.40_727.3490m/z | 2M+Na | C_21_H_24_N_2_O_3_ | 32.7 | 3.43 | 67.36 | Gelsevirine |
| 146 | 5.62_536.2053n | M+Na, M+NH4 | C_30_H_32_O_9_ | 39.4 | 1.34 | 98.7 | Gomisin G* |
| 147 | 5.62_631.1073m/z | 2M+Na | C_15_H_12_O_7_ | 32.3 | 2.4 | 64.21 | Taxifolin |
| 148 | 5.62_679.1499m/z | 2M+Na | C_14_H_16_O_9_ | 33.2 | 2.81 | 69.44 | Bergenin |
| 149 | 5.65_421.1764m/z | M+Na | C_22_H_26_N_2_O_5_ | 34.9 | 7.46 | 82.12 | 11,12-Methylenedioxykopsinaline |
| 150 | 5.65_463.1199m/z | M+Na | C_20_H_24_O_11_ | 27.9 | -2.75 | 42.75 | Ginkgolide C |
| 151 | 5.69_449.1169m/z | M+Na | C_15_H_26_N_2_O_10_S | 34.7 | -7.42 | 81.43 | N-Fructosyl gamma-glutamyl-S-Methylcysteine |
| 152 | 5.72_383.1493m/z | M+Na | C_20_H_24_O_6_ | 35.7 | 7.62 | 86.82 | 3,3'-Dimethoxy-8,9-epoxylignan-4,4',9'-triol |
| 153 | 5.72_823.2924m/z | 2M+Na | C_22_H_24_O_7_ | 34.1 | -1.48 | 72.15 | Schizanrin N |
| 154 | 5.76_925.3792m/z | 2M+H | C_24_H_30_O_9_ | 35.6 | -6.58 | 85.32 | 3,7-dihydroxy-1,2,13,14-tetramethoxydibenzocyclooctadiene 12-O-D-glucopyranoside |
| 155 | 5.79_297.1123m/z | 2M+H | C_9_H_8_O_2_ | 29.5 | 0.58 | 48.09 | p-Coumaraldehyde |
| 156 | 5.79_414.1684n | M+H, M+Na | C_23_H_26_O_7_ | 38.6 | 1.28 | 94.41 | Neokadsuranin |
| 157 | 5.79_687.2848m/z | M+H | C_32_H_46_O_16_ | 38.7 | -1.61 | 95.22 | Seco-isolariciresinol diglucoside |
| 158 | 5.82_514.2208n | M+Na, 2M+Na, M+NH4 | C_28_H_34_O_9_ | 39.1 | 1.03 | 96.92 | (-)-Tigloyl-deangeloyl-gomisin F |
| 159 | 6.28_287.0901m/z | M+H | C_16_H_14_O_5_ | 36.4 | -4.52 | 83.28 | Sakuranetin |
| 160 | 6.28_402.2045n | M+H, M+NH4, M+Na, 2M+Na | C_23_H_30_O_6_ | 38.6 | 0.66 | 93.64 | Gomisin K2* |
| 161 | 6.28_447.1766m/z | M+Na | C_25_H_28_O_6_ | 46.9 | -2.81 | 87.03 | Flavanone base + 4O, 2Prenyl |
| 162 | 6.28_489.2311m/z | 2M+H | C_15_H_16_O_3_ | 35.1 | 8.11 | 84.33 | Osthole |
| 163 | 6.38_530.3821m/z | M+NH4 | C_32_H_48_O_5_ | 37.2 | -3.62 | 90.45 | Acetoxycoccinic acid |
| 164 | 6.38_993.3792m/z | 2M+H | C_26_H_28_N_2_O_8_ | 38.8 | 2.78 | 97.48 | Tetrahydroalstonine |
| 165 | 6.42_1111.3739m/z | 2M+Na | C_28_H_32_O_11_ | 32.1 | -3.86 | 64.86 | Schizanrin G |
| 166 | 6.42_654.2913m/z | M+NH4 | C_35_H_40_O_11_ | 34.7 | 0.63 | 74.16 | Schisantherin J |
| 167 | 6.56_536.2052n | M+Na, 2M+Na, M+NH4 | C_30_H_32_O_9_ | 35.5 | 0.98 | 82 | Schisantherin A* |
| 168 | 6.56_654.2913m/z | M+Na | C_33_H_45_NO_11_ | 36.4 | 4.44 | 75.46 | Mesaconitine |
| 169 | 6.57_269.0447m/z | M+H | C_15_H_8_O_5_ | 36.3 | 0.97 | 73.31 | Coumestan base + 2O |
| 170 | 6.57_271.0599m/z | M+H | C_15_H_10_O_5_ | 35.5 | -0.75 | 78.5 | Baicalein |
| 171 | 6.57_374.2079n | M+H, M+NH4, M+Na, 2M+Na | C_22_H_30_O_5_ | 38.3 | -3.79 | 95.8 | (2S,3S)-4,4-bis (3,4-dimethoxyphenyl)-2,3-Dimethyl-1-butano |
| 172 | 6.57_414.1684n | M+H, M+Na, 2M+H | C_23_H_26_O_7_ | 38.9 | 1.21 | 95.98 | Kadsulignan L |
| 173 | 6.57_514.2207n | M+Na, 2M+Na, M+NH4 | C_28_H_34_O_9_ | 38.4 | 0.83 | 92.92 | Tigloylgomisin P |
| 174 | 6.57_594.1604n | M+H, M+Na, M+NH4 | C_27_H_30_O_1_5 | 33 | 3.28 | 68.25 | Apigenin-6-C-glucoside-7-O-glucoside |
| 175 | 6.57_616.3108m/z | M+H | C_33_H_45_NO_10_ | 38.5 | -1.28 | 93.81 | Hypaconitine |
| 176 | 6.61_259.1671m/z | M+Na | C_15_H_24_O_2_ | 38.4 | 1.19 | 93.19 | Dihydroxy-H-guaia-6,10(14)-dien |
| 177 | 6.61_279.0642m/z | M+Na | C_15_H_12_O_4_ | 36.2 | 5.6 | 84.17 | 2',4,4'-Trihydroxychalcone |
| 178 | 6.61_429.1886m/z | M+Na | C_22_H_30_O_7_ | 39.1 | 0.65 | 96.13 | Longipedlignan Q |
| 179 | 6.61_592.2782m/z | M+NH4 | C_30_H_38_O_11_ | 36.6 | 5.11 | 88.74 | Schisdilactone I |
| 180 | 6.71_543.1633m/z | M+Na | C_29_H_28_O_9_ | 38.3 | 1.39 | 93.41 | Schisantherin D* |
| 181 | 6.71_559.1378m/z | 2M+Na | C_16_H_12_O_4_ | 36 | 2.67 | 83.17 | Formononetin |
| 182 | 6.71_566.2388m/z | M+NH4 | C_31_H_32_O_9_ | 38.2 | 0.63 | 91.87 | Longipedlignan C |
| 183 | 6.71_677.0714m/z | 2M+Na | C_16_H_9_NO_7_ | 35.4 | 9.75 | 86.3 | Aristolochic acid C |
| 184 | 6.74_627.2892m/z | 2M+Na | C_18_H_22_O_4_ | 29.7 | -6.1 | 55.65 | Nordihydroguaiaretic acid |
| 185 | 6.78_364.1502m/z | M+Na | C_20_H_23_NO_4_ | 34.7 | -5.07 | 79.38 | Isocorydine |
| 186 | 6.81_516.2247m/z | M+NH4 | C_27_H_30_O_9_ | 37.6 | 3.7 | 92.53 | Kadsutherin F |
| 187 | 6.84_633.1451m/z | M+Na | C_27_H_30_O_16_ | 35.5 | 4.13 | 82.32 | Quercetin 3-O-[2''-O-b-D-glucopyranosyl]-A-L-rhamnopyranoside |
| 188 | 6.91_569.2395m/z | M+Na | C_29_H_38_O_10_ | 35.8 | 6.95 | 86.72 | 17-Hydroxyangeloylgomisin Q |
| 189 | 7.00_528.2352n | M+H, M+Na | C_29_H_36_O_9_ | 38.1 | -1.43 | 91.99 | schisandroside E |
| 190 | 7.00_659.4038m/z | 2M+H | C_20_H_27_NO_3_ | 38.8 | -2.53 | 97.07 | Hetisine |
| 191 | 7.10_371.1841m/z | M+H | C_22_H_26_O_5_ | 32.8 | -3.35 | 68.07 | (-) gomisin L2 |
| 192 | 7.10_402.2044n | M+H, M+Na, M+NH4, 2M+Na | C_23_H_30_O_6_ | 39.3 | 0.28 | 96.63 | Gomisin K1 |
| 193 | 7.10_605.0913m/z | 2M+H | C_15_H_10_O_7_ | 34.5 | -2.1 | 75.14 | Quercetin |
| 194 | 7.10_703.3436m/z | 2M+H | C_18_H_25_NO_6_ | 36.1 | -0.02 | 80.31 | Retrorsine |
| 195 | 7.20_425.1457m/z | M+H | C_20_H_24_O_1_0 | 38.1 | 3.53 | 92.76 | Ginkgolide B |
| 196 | 7.20_469.3311m/z | M+H | C_30_H_44_O_4_ | 39.2 | -0.21 | 96.16 | Changnanic acid |
| 197 | 7.20_516.2336m/z | M+NH4 | C_26_H_30_N_2_O_8_ | 38.5 | -0.83 | 92.84 | Strictosamide |
| 198 | 7.20_532.3992m/z | M+NH4 | C_32_H_50_O_5_ | 39.3 | -0.95 | 97.68 | Schinalactone D |
| 199 | 7.20_600.2453m/z | M+NH4 | C_31_H_34_O_11_ | 33.3 | 2.32 | 69.49 | Renchangianin A |
| 200 | 7.20_647.2393m/z | M+Na | C_36_H_36_N_2_O_8_ | 34.9 | 4.73 | 79.92 | Grossamide or its isomer (not validated) |
| 201 | 7.34_226.0688m/z | M+Na | C_9_H_17_NS_2_ | 28.1 | -3.19 | 43.92 | 7-Methylsulfenylheptyl isothiocyanate |
| 202 | 7.34_357.1696m/z | M+H | C_21_H_24_O_5_ | 38.2 | -0.27 | 91.09 | Arisantetralone C |
| 203 | 7.34_375.1821m/z | M+H | C_21_H_26_O_6_ | 37.5 | 4.92 | 93.32 | Longipedlignan R |
| 204 | 7.34_497.1572m/z | M+NH4 | C_22_H_23_O_12_ | 35 | 9.28 | 85.24 | Petunidin 3-galactoside |
| 205 | 7.34_759.3292m/z | 2M+Na | C_21_H_24_N_2_O_4_ | 35.2 | -9.77 | 86.82 | Formosanine |
| 206 | 7.56_359.1485m/z | M+H | C_20_H_22_O_6_ | 37.3 | -1.06 | 79.3 | Matairesinol |
| 207 | 7.56_523.2302m/z | M+Na | C_28_H_36_O_8_ | 38.6 | 0 | 92.8 | (R-biar)-12-angeloyloxy-6,7,8,9-tetrahydro-1,2,3,13,14-pentamethoxy-7,8-dimethyl-7 dibenzo [a,c] cyclooctenol |
| 208 | 7.63_568.2902m/z | M+NH4 | C_32_H_38_O_8_ | 35.4 | -0.46 | 77.43 | heteroclitalactone K |
| 209 | 7.66_1051.4287m/z | 2M+Na | C_28_H_34_O_9_ | 33.9 | -1.02 | 70.65 | Kadlongilignan F |
| 210 | 7.66_515.2279m/z | M+H | C_28_H_34_O_9_ | 39.6 | 0.69 | 99.05 | Schisantherin C |
| 211 | 7.66_536.2025n | M+H, M+Na | C_30_H_32_O_9_ | 38.3 | -4.01 | 96.4 | Gomisin C |
| 212 | 7.73_620.2519m/z | M+NH4 | C_34_H_34_O_10_ | 30.9 | 4.83 | 60.27 | Heteroclitin L |
| 213 | 7.95_471.2378m/z | M+H | C_27_H_34_O_7_ | 38.8 | 0.07 | 93.84 | Schisanchinins B |
| 214 | 7.99_386.1728n | M+H, M+Na, 2M+Na | C_22_H_26_O_6_ | 39.5 | -0.3 | 97.88 | (+)-Gomisin M1 |
| 215 | 7.99_653.4038m/z | 2M+H | C_20_H_26_N_2_O_2_ | 37.7 | -3.58 | 92.77 | Ajmaline |
| 216 | 8.02_481.1835m/z | M+Na | C_25_H_30_O_8_ | 39.1 | 0.56 | 96.32 | Longipedunin B |
| 217 | 8.02_497.1574m/z | M+NH4 | C_22_H_23_O_12_ | 35.7 | 9.59 | 88.98 | Petunidin-3-O-beta-glucoside |
| 218 | 8.02_612.3175m/z | M+NH4 | C_34_H_42_O_9_ | 34.2 | 1.33 | 72.67 | heteroclitalactone J |
| 219 | 8.05_634.2275m/z | M+NH4 | C_34_H_32_O_11_ | 37.2 | -1.27 | 87.67 | Heteroclitin O |
| 220 | 8.29_359.1483m/z | M+H | C_20_H_22_O_6_ | 35.4 | -1.82 | 79.3 | (+)-Pinoresinol |
| 221 | 8.29_386.1721n | M+H, M+Na, 2M+Na | C_22_H_26_O_6_ | 39 | -2.1 | 97.74 | (+)-Gomisin M2 |
| 222 | 8.29_595.1972m/z | M+H | C_28_H_34_O_14_ | 34.7 | -8.33 | 79.61 | isosakuranetin-7-O-neohesperidoside |
| 223 | 8.29_635.3335m/z | 2M+Na | C_20_H_22_N_2_O | 33.9 | -3.44 | 72.97 | Koumine |
| 224 | 8.36_529.1832m/z | M+Na | C_29_H_30_O_8_ | 39.4 | -0.24 | 97.11 | Schizarin E |
| 225 | 8.39_465.1519m/z | M+Na | C_24_H_26_O_8_ | 34.5 | -0.29 | 72.85 | Acetoxyloxokadsurane |
| 226 | 8.39_465.1519m/z | M+Na | C_24_H_26_O_8_ | 34.5 | -0.29 | 72.85 | Schiarisanrin B |
| 227 | 8.45_523.3053m/z | M+H | C_32_H_42_O_6_ | 38.7 | -0.27 | 93.86 | Schisanchinlactone B |
| 228 | 8.65_559.2159m/z | M+H | C_29_H_34_O_11_ | 33.6 | -2.66 | 71 | Schisantherin G |
| 229 | 8.72_317.2079m/z | M+NH4 | C_15_H_25_NO_5_ | 37.1 | 2.71 | 88.51 | Rinderine |
| 230 | 8.85_699.3139m/z | 2M+Na | C_20_H_22_N_2_O_3_ | 37.3 | -2.05 | 89.02 | Hydroxygardnutine |
| 231 | 8.89_495.1991m/z | M+Na | C_26_H_32_O_8_ | 37.6 | 0.42 | 88.53 | Isobutyroylbinankadsurin A |
| 232 | 8.94_505.1826m/z | M+Na | C_27_H_30_O_8_ | 34.3 | -1.44 | 73.1 | Heteroclitin D |
| 233 | 9.06_833.4501m/z | 2M+H | C_24_H_32_O_6_ | 37 | 3.66 | 89.41 | Schisandrin A* |
| 234 | 9.28_254.0583n | M+H, 2M+H, 2M+Na | C_15_H_10_O_4_ | 37.8 | 1.69 | 79.84 | Apigeninidin |
| 235 | 9.28_271.0598m/z | M+H | C_15_H_10_O_5_ | 38.3 | -1.04 | 92.99 | Isoflavonoids |
| 236 | 9.28_386.2082m/z | M+NH4 | C_21_H_24_N_2_O_4_ | 36.7 | 2.07 | 85.37 | Speciofiline |
| 237 | 9.28_442.2358n | M+NH4, M+Na | C_26_H_34_O_6_ | 38.5 | 0.51 | 93.27 | Cinobufagin |
| 238 | 9.28_534.1414n | M+Na, 2M+Na, 2M+H | C_25_H_26_O_13_ | 29.4 | 7.57 | 55.4 | Flavone base + 3O, C-Pen, C-Pen |
| 239 | 9.28_887.4185m/z | 2M+Na | C_24_H_32_O_7_ | 35.8 | -0.36 | 79.26 | Isoschisandrin |
| 240 | 9.31_339.1570m/z | M+H | C_21_H_22_O_4_ | 32.6 | -6.08 | 69.74 | Chalcone base + 2O, 1MeO, 1Prenyl or Licochalcone A (not validated) |
| 241 | 9.31_466.3086n | M+H, 2M+H | C_30_H_42_O_4_ | 39.1 | 0.6 | 96.24 | Kadsudilactone C |
| 242 | 9.31_851.2602m/z | 2M+Na | C_22_H_22_O_8_ | 36.8 | 9.77 | 94.72 | (-)-Podophyllotoxin |
| 243 | 9.35_371.1005m/z | M+Na | C_20_H_16_N_2_O_4_ | 38.6 | 0.74 | 91.76 | Camptothecin |
| 244 | 9.64_425.1591m/z | M+Na | C_22_H_26_O_7_ | 36.8 | 4.94 | 89.93 | Binankadsurin A |
| 245 | 9.64_507.1990m/z | M+Na | C_27_H_32_O_8_ | 39.3 | 0.15 | 96.86 | Angeloylgomisin M1 |
| 246 | 10.16_556.3261m/z | M+NH4 | C_32_H_42_O_7_ | 36.1 | -1.52 | 82.15 | Xuetonglactone C |
| 247 | 10.20_528.2951m/z | M+NH4 | C_30_H_38_O_7_ | 37.2 | -0.91 | 87.31 | Schinchinenins D |
| 248 | 10.42_555.2234m/z | M+Na | C_28_H_36_O_10_ | 33.9 | 6.33 | 76.93 | Wuweizidilactone H |
| 249 | 10.66_471.3463m/z | M+H | C_30_H_46_O_4_ | 38.6 | -1.2 | 94.34 | Nigranoic acid |
| 250 | 10.73_509.2135m/z | M+Na | C_27_H_34_O_8_ | 38.9 | -2.25 | 97.15 | Schisantherin F |
| 251 | 10.76_1005.2384m/z | 2M+H | C_24_H_22_O_12_ | 33.4 | 8.86 | 74.12 | Isoflavone base + 2O, O-MalonylHex |
| 252 | 10.76_1031.3813m/z | 2M+Na | C_26_H_32_O_10_ | 31.4 | -6.91 | 64.63 | 1,2,13,14-tetramethoxydibenzocyc looctadiene 3,12-O-D-diglucopyranoside |
| 253 | 10.76_1037.2253m/z | 2M+H | C_24_H_22_O_13_ | 24.7 | 5.69 | 29.91 | Isoflavone base + 3O, O-MalonylHex |
| 254 | 10.76_284.0696n | M+H, 2M+Na | C_16_H_12_O_5_ | 37.1 | 3.85 | 90.23 | Acacetin |
| 255 | 10.76_290.0927n | M+H, 2M+Na | C_16_H_12_O_5_ | 31.2 | 3.85 | 64.92 | Biochanin A |
| 256 | 10.76_339.1558m/z | M+H | C_21_H_22_O_4_ | 34.6 | -9.65 | 82.88 | Chalcone base + 2O, 1MeO, 1Prenyl or Licochalcone A |
| 257 | 10.76_400.1888n | M+H, M+Na, 2M+Na | C_23_H_28_O_6_ | 39.4 | 0.42 | 97.74 | Gomisin N* |
| 258 | 10.76_532.1168n | M+Na, 2M+H, M+H | C_25_H_24_O_13_ | 34.8 | -9.18 | 79.38 | Isoflavone base + 2O + 1MeO, O-MalonylHex |
| 259 | 10.76_535.1722m/z | M+Na | C_26_H_28_N_2_O_9_ | 35.6 | 6.75 | 85.25 | Lyalosidic acid |
| 260 | 10.76_595.2034m/z | M+H | C_28_H_34_O_14_ | 33.9 | 2.22 | 71.25 | 2',6'-Dihydroxy-4-methoxychalcone-4'-O-Neohesperid |
| 261 | 10.98_1111.4439m/z | 2M+Na | C_29_H_36_O_10_ | 32.9 | -6.44 | 72.05 | Schindilactone E |
| 262 | 11.25_445.1657m/z | M+Na | C_25_H_26_O_6_ | 40 | 8.27 | 75.13 | Flavone base + 4O, 2Prenyl |
| 263 | 11.29_228.0795n | M+H, M+Na, 2M+H | C_14_H_12_O_3_ | 37 | 3.69 | 79.63 | 3,4,5-Trihydroxystilbene |
| 264 | 11.29_339.1238m/z | M+NH4 | C_12_H_19_NO_7_S | 37.2 | 5.56 | 91.34 | N-Fructosyl alliin - H2O |
| 265 | 11.29_345.2061m/z | M+H | C_21_H_28_O_4_ | 37.9 | 0.29 | 89.82 | Monomethyl |
| 266 | 11.29_371.1847m/z | M+H | C_22_H_26_O_5_ | 38.4 | -1.63 | 94.1 | (-)-Gomisin L1 |
| 267 | 11.29_400.1885n | M+H, M+NH4, M+Na, 2M+Na | C_23_H_28_O_6_ | 39.2 | -0.29 | 96.29 | Schisandrin B* |
| 268 | 11.29_645.3389m/z | 2M+H | C_20_H_22_N_2_O_2_ | 26.3 | -7.17 | 39.18 | Gelsemine |
| 269 | 11.29_759.4085m/z | 2M+Na | C_22_H_28_N_2_O_3_ | 36.1 | -0.9 | 81.43 | Hirsutine |
| 270 | 11.29_975.2552m/z | 2M+Na | C_23_H_24_O_11_ | 28.6 | 2.42 | 45.06 | Flavone base + 2O, 2MeO, C-Hex |
| 271 | 11.32_479.2784m/z | M+H | C_30_H_38_O_5_ | 36.9 | -1.61 | 86.62 | Schisanlactone J |
| 272 | 11.32_514.2570n | M+Na, 2M+Na, M+NH4 | C_29_H_38_O_8_ | 39.3 | 0.71 | 97.38 | Kadsufolin A |
| 273 | 11.32_539.1932m/z | M+H | C_29_H_30_O_10_ | 36.4 | 3.68 | 86.09 | Kadsuphilin F |
| 274 | 11.32_557.1664m/z | M+H | C_28_H_28_O_12_ | 36.6 | 1.88 | 84.65 | Smiglaside C |
| 275 | 11.32_577.2382m/z | M+Na | C_22_H_42_N_4_O_8_S_2_ | 33.1 | 8.25 | 74.57 | D-Pantethine |
| 276 | 11.41_397.1644m/z | M+Na | C_21_H_26_O_6_ | 37.5 | 5.87 | 94.11 | Schinlignins B |
| 277 | 11.90_498.2842m/z | M+NH4 | C_29_H_36_O_6_ | 37 | -1.72 | 86.94 | Kadlongilactone D |
| 278 | 11.94_241.0863m/z | M+H | C_15_H_12_O_3_ | 36.7 | 1.67 | 84.37 | 2'-Hydroxyflavanone |
| 279 | 11.94_293.1134m/z | M+Na | C_17_H_18_O_3_ | 35.8 | -5.12 | 84.74 | E-Resveratrol trimethyl ether |
| 280 | 11.94_543.1994m/z | M+Na | C_30_H_32_O_8_ | 39.6 | 0.72 | 98.94 | Benzoylisogomisin O |
| 281 | 11.94_757.2042m/z | M+H | C_36_H_36_O_18_ | 31 | 8.98 | 64.75 | Quercetin 3-O-[2''-O-(6'''-O-p-coumaroyl)-b-D-glucopyranosyl]-a-L-rhamnopyranoside |
| 282 | 12.04_548.2293m/z | M+NH4 | C_31_H_30_O_8_ | 37.3 | 2.61 | 89.67 | Schiarisanrin D |
| 283 | 12.07_457.1829m/z | M+Na | C_23_H_30_O_8_ | 38.6 | -0.85 | 93.91 | kadlongilignan B |
| 284 | 12.07_484.2463n | M+H, M+NH4, 2M+Na | C_28_H_36_O_7_ | 39.4 | 0.38 | 97.54 | Angeloyl-(+)-gomisin K3* |
| 285 | 12.07_507.2361m/z | M+Na | C_28_H_36_O_7_ | 39.4 | 1.66 | 99.1 | Xuetongdilactone E |
| 286 | 12.07_528.2943m/z | M+NH4 | C_30_H_38_O_7_ | 36.7 | -2.42 | 86.46 | Longipedlactone G |
| 287 | 12.07_530.3113m/z | M+NH4 | C_30_H_40_O_7_ | 39.6 | 0.17 | 98.37 | Schinchinenins E |
| 288 | 12.07_556.3268m/z | M+NH4 | C_32_H_42_O_7_ | 33.8 | -0.18 | 69.35 | Heteroclitalactone L |
| 289 | 12.17_1051.4322m/z | 2M+Na | C_28_H_34_O_9_ | 35.7 | 2.3 | 81.06 | Gomisin B |
| 290 | 12.17_323.1267m/z | M+H | C_20_H_18_O_4_ | 38.7 | -3.44 | 89.02 | Flavone base + 2O, 1Prenyl |
| 291 | 12.17_357.1335m/z | M+H | C_20_H_20_O_6_ | 39.3 | 0.56 | 97.34 | Pluviatolide |
| 292 | 12.17_439.1721m/z | M+Na | C_23_H_28_O_7_ | 38 | -1.61 | 91.83 | Epigomisin O |
| 293 | 12.17_498.2258n | M+Na, 2M+Na | C_28_H_34_O_8_ | 38.4 | 0.87 | 93.19 | Angeloylisogomisin O* |
| 294 | 12.17_572.3223m/z | M+NH4 | C_32_H_42_O_8_ | 38.2 | 0.89 | 92.13 | Longipedlactone H |
| 295 | 12.17_611.3370m/z | 2M+Na | C_19_H_22_N_2_O | 28.8 | 2.23 | 46.19 | Koumidine |
| 296 | 12.60_329.1769m/z | M+H | C_20_H_24_O_4_ | 37.5 | 6.71 | 95.05 | Anwuligan* |
| 297 | 12.60_355.1539m/z | M+H | C_21_H_22_O_5_ | 40.6 | -0.26 | 93.16 | Chalcone base + 3O, 1MeO, 1Prenyl |
| 298 | 12.60_372.1572n | M+H, M+Na | C_21_H_24_O_6_ | 46.3 | -0.2 | 97.38 | Arctigenin |
| 299 | 12.60_384.1557n | M+H, M+Na, 2M+Na | C_22_H_24_O_6_ | 38 | -4.19 | 94.98 | Schisandrin C* |
| 300 | 12.96_273.0750m/z | M+H | C_15_H_12_O_5_ | 37 | -2.73 | 83.97 | Naringenin |
| 301 | 12.96_520.2100n | M+Na, 2M+Na, M+NH4 | C_30_H_32_O_8_ | 39.7 | 0.6 | 99.09 | Benzoylgomisin O* |
| 302 | 12.96_913.5078m/z | M+H | C_47_H_76_O_17_ | 36.3 | -8.43 | 90.91 | Soyasapogenol B base + O-HexA-Pen-dHex |
| 303 | 13.06_505.1838m/z | M+H | C_29_H_28_O_8_ | 34.4 | -3.68 | 76.48 | Interiotherin A* |
| 304 | 13.19_253.0858m/z | M+Na | C_14_H_14_O_3_ | 33.5 | 9.94 | 76.43 | Dihydroresveratrol; 3,4',5-Trihydroxybibenzyl |
| 305 | 13.19_254.0588n | M+H, M+Na, M+NH4 | C_15_H_10_O_4_ | 35 | 3.61 | 74.34 | 7,4'-Dihydroxyflavone |
| 306 | 13.19_291.0985m/z | M+H | C_11_H_18_N_2_O_5_S | 37.3 | -8.51 | 74.98 | Glutamyl-S-allylcysteine |
| 307 | 13.19_357.1336m/z | M+H | C_20_H_20_O_6_ | 39.2 | 0.81 | 96.9 | Piperitol |
| 308 | 13.19_369.1689m/z | M+Na | C_20_H_26_O_5_ | 37.9 | 4.71 | 94.86 | (2S,3S)-4,4-bis (4-hydroxy-3-methoxyphenyl)-2,3-dimethyl-1-butanol |
| 309 | 13.19_439.1723m/z | M+Na | C_23_H_28_O_7_ | 38 | -1.05 | 91.08 | Gomisin O |
| 310 | 13.19_455.1489m/z | M+H | C_28_H_22_O_6_ | 38.5 | -0.13 | 91.65 | Epsilon-Viniferin |
| 311 | 13.19_498.2257n | M+Na, 2M+Na, M+NH4 | C_28_H_34_O_8_ | 39.7 | 0.71 | 99.25 | Angeloylgomisin O* |
| 312 | 13.38_475.2306m/z | M+H | C_26_H_34_O_8_ | 38.3 | -4.28 | 96.34 | Kadsufolin B |
| 313 | 13.62_527.1681m/z | M+Na | C_29_H_28_O_8_ | 39.5 | 0.99 | 98.62 | Benzoyloxokadsurane |
| 314 | 13.62_550.2436m/z | M+NH4 | C_31_H_32_O_8_ | 39.4 | 0.05 | 97.04 | Kadsufolin C |
| 315 | 13.79_296.0748m/z | M+Na | C_11_H_15_NO_7_ | 37.1 | 2.8 | 86.89 | Pentose + Proline |
| 316 | 13.82_353.1383m/z | M+H | C_21_H_20_O_5_ | 41.8 | -0.1 | 95.85 | Isoflavone base + 2O, 1MeO, 1Prenyl |
| 317 | 13.82_571.2826m/z | 2M+H | C_17_H_19_NO_3_ | 35.2 | 4.1 | 80.62 | Piperine |
| 318 | 13.82_641.1114m/z | 2M+H | C_15_H_12_O_8_ | 37.5 | -3.53 | 91.15 | Flavanone base + 6O |
| 319 | 14.15_514.2802m/z | M+NH4 | C_29_H_36_O_7_ | 39.6 | 0.63 | 98.99 | kadlongilactone F |
| 320 | 14.15_653.3302m/z | 2M+H | C_19_H_22_N_2_O_3_ | 35.3 | -4.87 | 82.15 | Gelsenicine |
| 321 | 14.51_629.2581m/z | 2M+Na | C_19_H_17_N_3_O | 36.1 | -9.03 | 89.7 | Evodiamine |
| 322 | 14.61_588.3567m/z | M+H | C_33_H_49_NO_8_ | 33.8 | 6.21 | 75.48 | Pseudojervine |
| 323 | 14.64_576.3555m/z | M+NH4 | C_32_H_46_O_8_ | 38.5 | 4.32 | 97.35 | Xuetongdilactone A |
| 324 | 14.68_553.2779m/z | M+H | C_32_H_40_O_8_ | 37.5 | -3.1 | 91.35 | heteroclitalactone I |
| 325 | 15.46_553.2203m/z | M+Na | C_27_H_34_N_2_O_9_ | 35 | 8.7 | 84.8 | Strictosidine |
| 326 | 16.29_470.3383n | M+H, M+Na | C_30_H_46_O_4_ | 37.5 | -2.8 | 90.73 | Kadsuric acid |
| 327 | 16.41_535.3417m/z | M+Na | C_32_H_48_O_5_ | 37.4 | 4.57 | 92.22 | schisanhenric acid |
| 328 | 16.75_264.2447n | M+H, M+NH4 | C_18_H_32_O | 38.9 | -2.28 | 97.15 | guai-en-ol |
| 329 | 16.79_655.2379m/z | M+Na | C_32_H_40_O_13_ | 33 | 2.88 | 68.37 | Longipedunculatin A |
| 330 | 16.98_175.1237m/z | M+H | C_11_H_14_N_2_ | 39.4 | 3.91 | 92.54 | Gramine |
| 331 | 16.98_429.3738m/z | M+H | C_29_H_48_O_2_ | 33.9 | 2.53 | 72.48 | 7-oxositosterol |
| 332 | 16.98_637.2934m/z | M+H | C_38_H_40_N_2_O_7_ | 37.9 | 4.06 | 92.04 | Thalsimine |

Table S2. Linearity for ten lignans.

| Compound | Calibration curve | R^2^ | Linear range (μg/ml) |
| --- | --- | --- | --- |
| Schisandrol A | y = 26.0512 x - 142.8332 | 0.9996 | 7.50-480.00 |
| Gomisin D | y = 13.8223x - 9.3041 | 0.9996 | 1.56-100.00 |
| Gomisin J | y = 15.3123 x - 0.5437 | 1.0000 | 1.56-100.00 |
| Schisandrol B | y = 25.24 x + 12.4358 | 0.9996 | 3.75-240.00 |
| Angeloylgomisin H | y = 29.2581 x - 13.9034 | 1.0000 | 1.88-120.00 |
| Gomisin G | y = 18.7446 x + 6.8038 | 0.9996 | 1.09-70.00 |
| Schisantherin A | y = 23.4135 x - 2.9819 | 1.0000 | 0.55-70.00 |
| Schisandrin A | y = 24.3145 x + 25.1295 | 0.9996 | 1.10-140.00 |
| Schisandrin B | y = 27.6598 x + 7.5727 | 0.9998 | 2.74-350.00 |
| Schisandrin C | y = 25.1464 x - 8.2434 | 1.0000 | 1.41-90.00 |

**Anti-inflammatory activities method**

Cell culture and the determination of cell viability is the first step to assay anti-inflammatory activities of different parts. The RAW 264.7 macrophages was presented by researcher Sun Xiaobo of Institute of medicinal plants, the Dulbecco’s modified Eagle’s medium (DMEM) with 10 % fetal bovine serum (FBS) and 1.0 % penicillin-streptomycin solution were added in the cell which was cultured with 5% CO_2_ at 37℃. To test the anti-inflammatory activities, CCK-8 assay was used to detect the cell viability. The RAW264.7 cells were seeded into 96-well plates with a concentration of 2×10^6^ cells/mL and cultured at 37 ℃ in 5% CO_2_ for 24 h before test. Then, 100μL different concentrations extracts (25, 50, 100, 200 μg/mL) from Roots, Stems, Leaves and Fruits in *S. chinensis* were added to the culture plate and incubator for 24 hours, next, 10μL CCK-8 solution was add to each well, incubate for 1.5h once again, and determine the absorbance at 450nm with an enzyme labeling instrument. At least 3 repetitions were set in each group, the results were expressed by the mean value, and the cell viability was computed according to the following formula:

Cell viability (%) = (A1-A_blank_) / (A0 -A_blank_) × 100%

(A1: absorbance of pores with cells, CCK-8 solution and drug solution; A_blank_: absorbance of pores with culture medium and CCK-8 solution; A0: absorbance of pores with cells and CCK-8 solution).

The NO standard curve was drawn and determined by Griess kit. Griess Reagent Kit uses a classic protocol for the estimation of nitrite in biological samples. Nitrite is reduced to nitrogen oxide using Griess Reagent I. Nitrogen oxide then reacts with Griess Reagent II forming a stable product that can be detected by its absorbance at 540 nm. The two-step assay is simple, fast and can detect nitrite levels as low as 1 nmol/well. The specific methods are as follows: Dilute the standard (1-100μΜ) with serum-free DMEM medium. Dilution process of standard: take 10 μL 1m NaNO2 solution, add 1ml serum-free DMEM medium to dilute 100 times to obtain 10mm solution, and take 10 μ L and 1ml serum-free DMEM medium were diluted 100 times to obtain 100 μM solution, half diluted, prepared with concentration gradients of 50, 25, 12.5, 6.25, 3.125 and 0 μM solution, add 95% to each well μL standard, 50 μL Griess reagent Ⅰ and 50 μL Griess reagent Ⅱ, after 15min, measure the absorbance at 540nm and calculate the standard curve.
